# Circulating serum profile of small non-coding RNAs in patients with anaphylaxis beyond microRNAs

**DOI:** 10.3389/falgy.2024.1307880

**Published:** 2024-02-07

**Authors:** Sergio Fernández-Bravo, Diana Betancor, Javier Cuesta-Herranz, Pablo Rodríguez del Río, María Dolores Ibañez-Sandín, Emilio Nuñez-Borque, Vanesa Esteban

**Affiliations:** ^1^ Department of Allergy and Immunology, IIS-Fundación Jiménez Díaz, UAM, Madrid, Spain; ^2^ Allergy Department, Hospital Universitario Fundación Jiménez Díaz, Madrid, Spain; ^3^ Allergy Department, Hospital Infantil Universitario Niño Jesús, Fundación HNJ, IIS-P, Madrid, Spain; ^4^ Faculty of Medicine and Biomedicine, Alfonso X El Sabio University, Madrid, Spain

**Keywords:** anaphylaxis, biomarkers, drug allergy, food allergy, sncRNA

## Abstract

**Introduction:**

Anaphylaxis is the most severe manifestation of allergic disorders. Currently, an increasing number of cells, pathways and molecules involved in the etiopathogenesis of anaphylaxis are being discovered. However, there are no conclusive biomarkers to confirm its diagnosis. Small non-coding RNAs (sncRNAs) are 18-200 nucleotide molecules that can be divided into: microRNAs (miRNAs), Piwi-interacting RNAs (piRNAs), small nucleolar RNAs (snoRNAs), small nuclear RNAs (snRNAs), transference RNA derived fragments (tRFs) and YRNA derived fragments (YRFs). These molecules participate in cell-cell communication modulating various physiological processes and have been postulated as non-invasive biomarkers of several pathologies. Therefore, in this study we characterized the serum circulating profile of other sncRNA beyond miRNAs in two populations of 5 adults and 5 children with drug- and food-mediated anaphylaxis, respectively.

**Methods:**

Samples were obtained from each patient under two different conditions: during anaphylaxis and 14 days after the reaction (control). The sncRNA analysis was carried out by Next Generation Sequencing (NGS).

**Results:**

A total of 671 sncRNAs (3 piRNAs, 74 snoRNAs, 54 snRNAs, 348 tRFs and 192 YRFs) were identified in adults with drug-induced anaphylaxis, while 612 sncRNAs (2 piRNAs, 73 snoRNAs, 52 snRNAs, 321 tRFs and 164 YRFs) were characterized in children with food-mediated anaphylaxis. However, only 33 (1 piRNA, 4 snoRNAs, 1 snRNAs, 7 tRFs and 20 YRFs) and 80 (4 snoRNAs, 6 snRNAs, 54 tRFs and 16 YRFs) of them were statistically different between both conditions, respectively. Among them, only three (Y_RNA.394, Y_RNA.781 and SCARNA2) were common to both adults and children analysis.

**Discussion:**

This study provides a differential profile of circulating serum sncRNAs beyond miRNAs in patients with anaphylaxis, postulating them as candidate biomarkers for this pathological event and as novel mediators of the reaction.

## Introduction

Anaphylaxis is the most severe manifestation of allergic disorders and represents a fast-developing systemic hypersensitivity reaction that can lead to the patient's death. This pathological event can be caused by exposure to several allergens, among which the most common are foods, drugs, and Hymenoptera venoms (1). Etiologic distribution of anaphylaxis differs according to age, being drugs the most common triggers in adults, whereas foods are the main triggers in children (2, 3). These allergens result in the activation of the effector cells of the reaction causing the signs and symptoms of this pathological event. Among these, skin and mucosa are the most frequently affected (80%–90%), followed by the respiratory (70%), cardiovascular and gastrointestinal (45%), and nervous (15%) systems (4).

Currently, anaphylaxis is considered underdiagnosed because its diagnosis is based on the recognition of patients' clinical symptoms, which can be presented in many other pathologies (2, 5). In addition, the main biomarker used in clinical practice, serum tryptase, is not elevated in most cases (6, 7). Both, the absence of unique symptoms and accurate biomarkers, result in the failure to identify this pathological event and, therefore, to manage it optimally (2, 5).

Mechanistically, among the different molecular mechanisms involved in anaphylaxis, the IgE-mediated pathway is the main one described in humans. It comprises two phases: a first sensitization phase, after exposure to the allergen, and a second effector phase, in which the effector cells are activated after re-exposure to the antigen leading to the anaphylactic reaction (8). However, in some patients, no detectable specific IgE levels against the allergen inducing anaphylaxis are observed. In contrast, others present elevated values of this molecule without developing any symptom (9–11). Therefore, it has been suggested that there exists other signaling pathways independent or complementary to IgE participating in the reaction (8, 12). Precisely, over time, more molecules and mediators involved in the underlying molecular mechanisms of anaphylaxis have been identified (13).

Non-coding RNA (ncRNA) were considered for many years as “junk” DNA, although they have been found to be functional. This ncRNA can be classified by its length in two groups: small-ncRNAs (sncRNAs), when they have 18–200 nucleotides, and long-ncRNAs (lncRNAs), when they have >200 nucleotides. In turn, sncRNAs can be divided into microRNAs (miRNAs), Piwi-interacting RNAs (piRNAs), small nucleolar RNAs (snoRNAs), small nuclear RNAs (snRNAs), transference RNA derived fragments (tRFs) and YRNA derived fragments (YRFs) (14). Among them, the most studied are microRNAs, although knowledge of the rest of sncRNAs has increased in recent years. These molecules participate in cell–cell communication modulating various physiological processes and have been related to epigenetic regulation, DNA stability, and transcription and translation control (14, 15). Moreover, sncRNAs are very stable, resistant to degradation and highly correlated with the pathological state. Thus, they have been postulated as non-invasive biomarkers of several pathologies (16). Precisely, MacGrath et al. recently demonstrated an increase of snoRNAs in patients with severe anaphylaxis compared to other systemic inflammatory processes, proposing them as diagnostic molecular markers of these episodes (17).

The aim of this study was to identify the circulating serum profile of sncRNAs, beyond miRNAs, in children with food-mediated anaphylaxis and adults with drug-mediated anaphylaxis.

## Materials and methods

### Study design

This descriptive and exploratory study was conducted in patients with anaphylaxis recruited from four Spanish hospitals (Fundación Jiménez Díaz University Hospital, Ramón y Cajal Hospital, Guadalajara's University Hospital and Niño Jesús University Children's Hospital) between September 2016 and September 2018.

### Ethical committee

The protocol was approved by the relevant Ethics Committee (CEIm FJD, PIC057-19 and PIC166-22_FJD). Authors adhered to the declaration of Helsinki and all patients were included after giving informed consent by the donors or their relatives. Inclusion criteria were acceptance to participate in the study and an objective diagnosis of the reaction. Exclusion criteria included the presence of any blood-borne disease or any psychic/psychological pathology that would condition the acceptance for the study.

### Patients

The experimental design included 20 paired sera samples from 10 patients with anaphylaxis well clinically characterized: 5 children with food-mediated anaphylaxis and 5 adults with drug-mediated anaphylaxis.

Diagnosis was confirmed by an allergist in agreement with the definition of anaphylaxis established by the “National Institute of Allergy and Infectious Disease and Food Allergy and Anaphylaxis Network” (18). From each patient, their gender, age, the trigger of the reaction, their signs and symptoms and the severity of the reaction, according to the criteria established by Brown (19), were recorder. In addition, serum tryptase levels were measured at the Fundación Jiménez Díaz Hospital using ImmunoCAP Phadia 1000 (Thermo Scientific).

### Sample collection

Serum samples were obtained from each patient under 2 conditions: during the acute phase (anaphylaxis) and at basal phase (control), at least 14 days after the reaction. Considering the subjects' heterogeneity, the basal phase of each participant was used as a control for its respective acute sample.

Peripheral blood was processed using tubes with separator gel (BD Vacutainer). After obtaining the sample, it was centrifuged at 1,200 g for 10 min at 4 °C. Subsequently, the serum was aliquoted in 0.5 ml and stored at −80 °C until its use.

### Circulating serum profile of small non-coding RNA by next-generation sequencing

All procedures were performed at Qiagen Genomics Service. The 10 samples evaluated by next-generation sequencing (NGS) from each group of patients were analyzed in the same batch and under identical conditions. In turn, in previous studies (20, 21), we confirmed the correct performance of the NGS by analyzing different quality controls.

Total RNA was isolated from 200 μl of serum using the miRNeasy Serum/Plasma Kit (Qiagen) according to manufacturer's instructions.

Library preparation was performed with the QIAseq miRNA Library Kit (Qiagen) from 5 μl of the isolated RNA. The RNA was retrotranscribed to copy DNA (cDNA), amplified by PCR (22 cycles) and purified from the samples. The libraries used were specific for small RNA species ranging from 15 to 40 nucleotides and were performed by a gel-free system through the addition of Unique Molecular Identifiers (UMIs). Quality controls of the library were determined using the Bioanalyzer 2100 or the TapeStation 4200 (Agilent Technologies). In addition, levels of sncRNAs were normalized to equimolar ratios depending on the quality of the library, the quality of the inserts and the concentration measurements. Subsequently, libraries were quantified by quantitative PCR (qPCR) and sequenced on an Illumina NextSeq500 instrument (10–12 million reads per sample).

The raw data were demultiplexed and FASTQ files were generated for each sample using the bcl2fastq program (Illumina Inc.). In turn, Cutadapt (1.11) software was used to remove adapter sequences and collapse the reads by UMIs with an internal script. Finally, these reads were mapped using the Bowtie2 program (2.2.2). The criterion established for this mapping was a perfect match with the sequences of the technique controls or with the miRbase_20 database. No errors or more than 1 mismatch were allowed during genome mapping.

### Statistical analysis

Data obtained by NGS were analyzed using the R 3.5.3 software. Principal component analysis (PCA) and heat maps were performed through the ClustVis web tool (https://biit.cs.ut.ee/clustvis/) (22). Volcano plots and graphical representations were carried out in the Graph Pad Prism 8 program. In the analysis of patients' characteristics, categorical variables were described as the frequency and percentage, while continuous data were presented as the mean ± standard error of the mean (SEM).

Normalization and relative quantification of NGS values were performed with the Prostar package (http://live.prostar-proteomics.org/) distributed by Bioconductor and implemented in R. The abundance data were transformed (log_2_) using R 3.5.3 software to obtain a symmetrical distribution prior to statistical analysis. In addition, all those sncRNAs that were not detected in at least two samples per condition were discarded. The matrices were normalized with the Cyclic Loess method because it decreases systemic variances and increases the efficiency in detecting changes in sncRNAs levels (23). Subsequently, Student's paired *t*-test was used to determine the significantly differential levels between the two conditions (anaphylaxis vs. control), as it classifies molecules based on their expression minimizing biases in the data (24). However, due to variations between patients and non-detection in some of the samples, few sncRNAs were detected with False Discovery Ratio (FDR), so this criterion was not used for statistical filtering. The threshold for statistical significance was established at *p* ≤ 0.05.

## Results

### Characteristics of the studied population

The clinical characteristics of the 10 patients with anaphylaxis used for the sncRNAs study are detailed in Table 1. This population presented an age ranged between 5 and 59 years old (32.6 ± 6.9) and less than half were female (40%). Specifically, the age of children was ranged between 5 and 15 years old (12.4 ± 1.9) and more than half were female (60%). In contrast, in the adult group, the age was ranged from 46 to 59 years old (52.8 ± 2.6) and only one woman was included (20%). In turn, all children presented food-mediated anaphylaxis, while in adults all reactions were caused by drugs, being nonsteroidal anti-inflammatory drugs (NSAIDs) the most frequent trigger (60%). On the other hand, half of the events were classified as moderate (Grade 2) and the other half as severe (Grade 3). However, severe anaphylaxis was more frequent in the adult cohort (60%) than in the children group (40%). Among the different manifestations, cutaneous (100%) and respiratory (90%) were the most frequent, followed by digestive (60%), cardiovascular (50%) and nervous (50%). Precisely, adults presented a higher percentage of cardiovascular and nervous symptoms (60%) compared to children (40%). However, all children showed respiratory manifestations (100%), while adults showed a lower percentage (80%). In addition, an increase in serum tryptase levels was observed in samples obtained during anaphylaxis (9.4 ± 2.1) compared to those used as control (4.0 ± 0.5). Specifically, this increase was higher in adults (12.1 ± 3.9 in anaphylaxis and 4.0 ± 0.7 in control samples) than in children (6.8 ± 1.1 in anaphylaxis and 4.1 ± 0.9 in control samples).

**Table 1:** Clinical characteristics of patients with anaphylaxis used for the sncRNAs study.

Patients			Triggers		Symptoms					Signs		Severity	Tryptase (ng/mL)	
N	Age	Gender	Group	Allergen	Skin	Digestive	Respiratory	Nervous	Vascular	Hr	SatO_2_	Grade	Anaphylaxis	Control
1	14	F	Food	Egg	Er, Pr, Ag	N, Ap	Rh	Dz	Hy	56	99%	3	2.6	2.29
2	15	M	Food	Cheese	Er		Rh, Wh			57	96%	2	8.03	5.69
3	5	M	Food	Milk	Er	Ap, V	Dy		Hy	85	94%	3	7.83	4.97
4	14	F	Food	Nut	Ur, Ag		Cg	Dz		96	99%	2	6.63	5.65
5	14	F	Food	Milk	Ur	N, Ap	Rh, Wh			107	97%	2	8.74	1.72
6	56	M	Drug	NSAID	Er, Pr, Cj	N	Dy			93	93%	2	8.47	4.07
7	46	F	Drug	NSAID	Er, Pr, Ur		Rh, Wh	Dz	Hy	–	–	3	27.2	3.45
8	47	M	Drug	*β*-lactam	Ur				Sy	78	89%	3	9.8	6.77
9	59	M	Drug	NSAID	Er, Pr	N, Ap, V, Dr	Dy	Dz, Hd		80	93%	2	5.43	2.59
10	56	M	Drug	Chemotherapy	Pr, Ag	N, Ap	Wh	Dz	T, Hy	135	92%	3	9.55	3.12

Gender, M (male), F (female); Trigger, NSAID (nonsteroidal anti-inflammatory drug); Skin, Er (erythema), Ur (urticaria), Pr (pruritus), Ag (angioedema), Cj (conjunctivitis); Digestive, N (nausea), V (vomit), Dr (diarrhea), Ap (abdominal pain); Respiratory, Dy (dyspnea), Wh (wheezing), Rh (rhinitis), Cg (cough); Nervous, Dz (dizziness), Hd (headache); Vascular, Hy (hypotension), T (tachycardia), Sy (syncope); Signs, Hr (heart rate), SatO_2_ (oxygen saturation).

### Small non-coding RNAs characterization in patients with anaphylaxis

**Figure 1:**
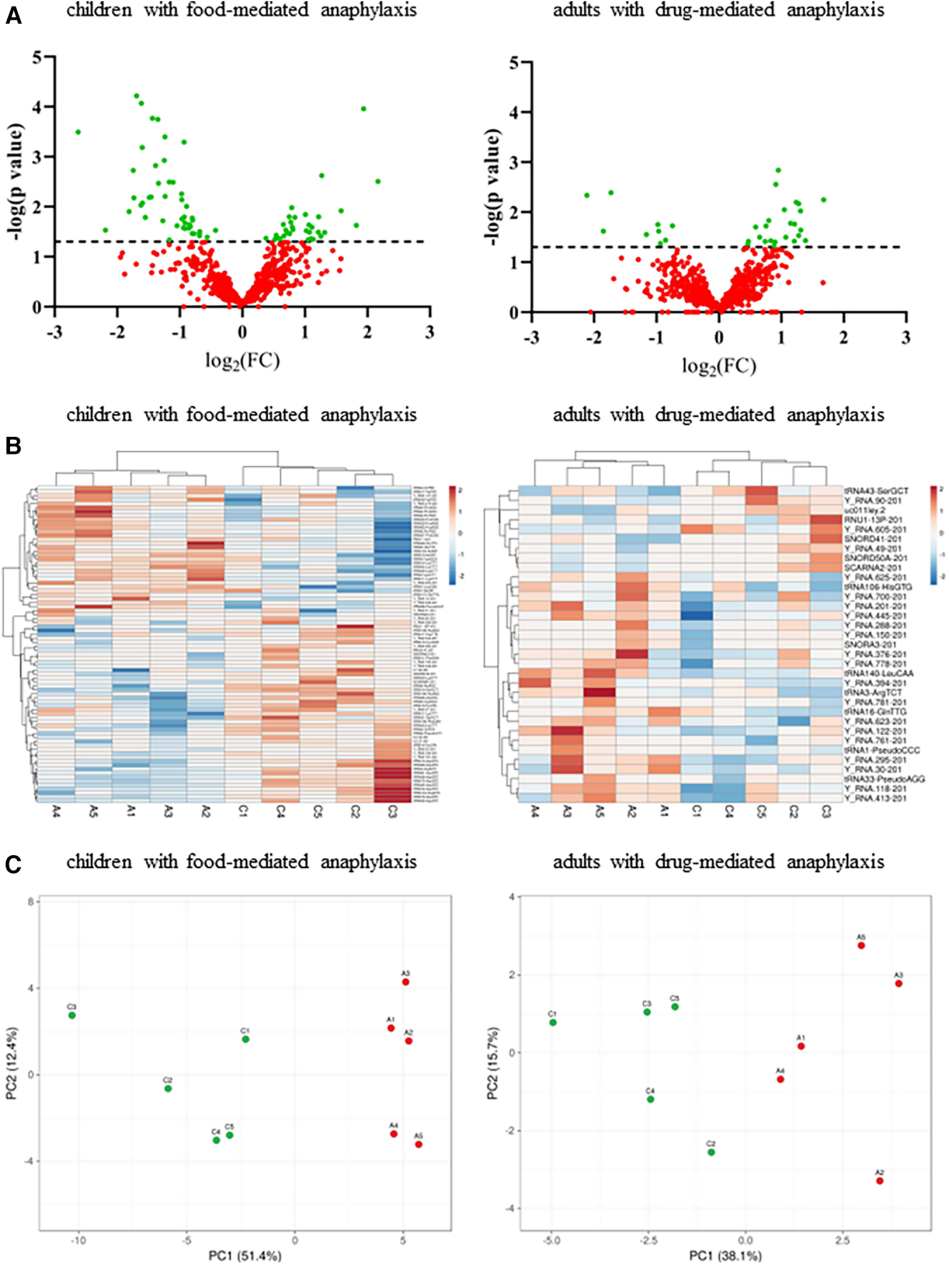
Analysis of circulating serum sncRNA profiles of patients with anaphylaxis. A: anaphylaxis samples, C: control samples. (**A**) Dispersion of all sncRNAs identified by NGS in the 5 children with food-mediated anaphylaxis (left panel) and the 5 adults with drug-mediated anaphylaxis (right panel). Green color shows statistically significant sncRNAs (*p* ≤ 0.05) between both conditions, while red color indicates those non-significant (*p* > 0.05). The line marks the significance threshold (*p* ≤ 0.05). FC, fold change (anaphylaxis/control). (**B**) Heat map representation of the statistically significant sncRNAs levels in each sample. The left panel shows the results of the 5 children with food-mediated anaphylaxis and the right panel revealed those of the 5 adults with drug-mediated anaphylaxis. Red color represents the increase, while blue color indicates the decrease during anaphylaxis. (**C**) Similarity between biological replicates and separation between anaphylaxis (red points) and control (green points) conditions in the 5 children with food-mediated anaphylaxis and the 5 adults with drug-mediated anaphylaxis. PC, principal component.

Serum levels of other sncRNAs were determined by NGS in a paired manner from the samples of 10 patients with anaphylaxis. A total of 612 sncRNAs were identified in children with food-mediated anaphylaxis (Supplementary Table S1), while 671 sncRNAs were described in adults with drug-mediated anaphylaxis (Supplementary Table S2). However, after statistical analysis, only 80 and 33 of them showed significant differences between both conditions, respectively (Figure 1A). Specifically, 33 increased and 47 decreased during anaphylaxis in children with food-mediated reactions. Instead, in adults with drug-mediated anaphylaxis, 24 increased and 9 decreased during the reaction (Figure 1B). Moreover, the similarity among the biological replicates and the separation between anaphylaxis and control conditions was verified by the PCA (Figure 1C).

### Small non-coding RNA clustering in children with food-mediated anaphylaxis and adults with drug-mediated anaphylaxis

**Figure 2:**
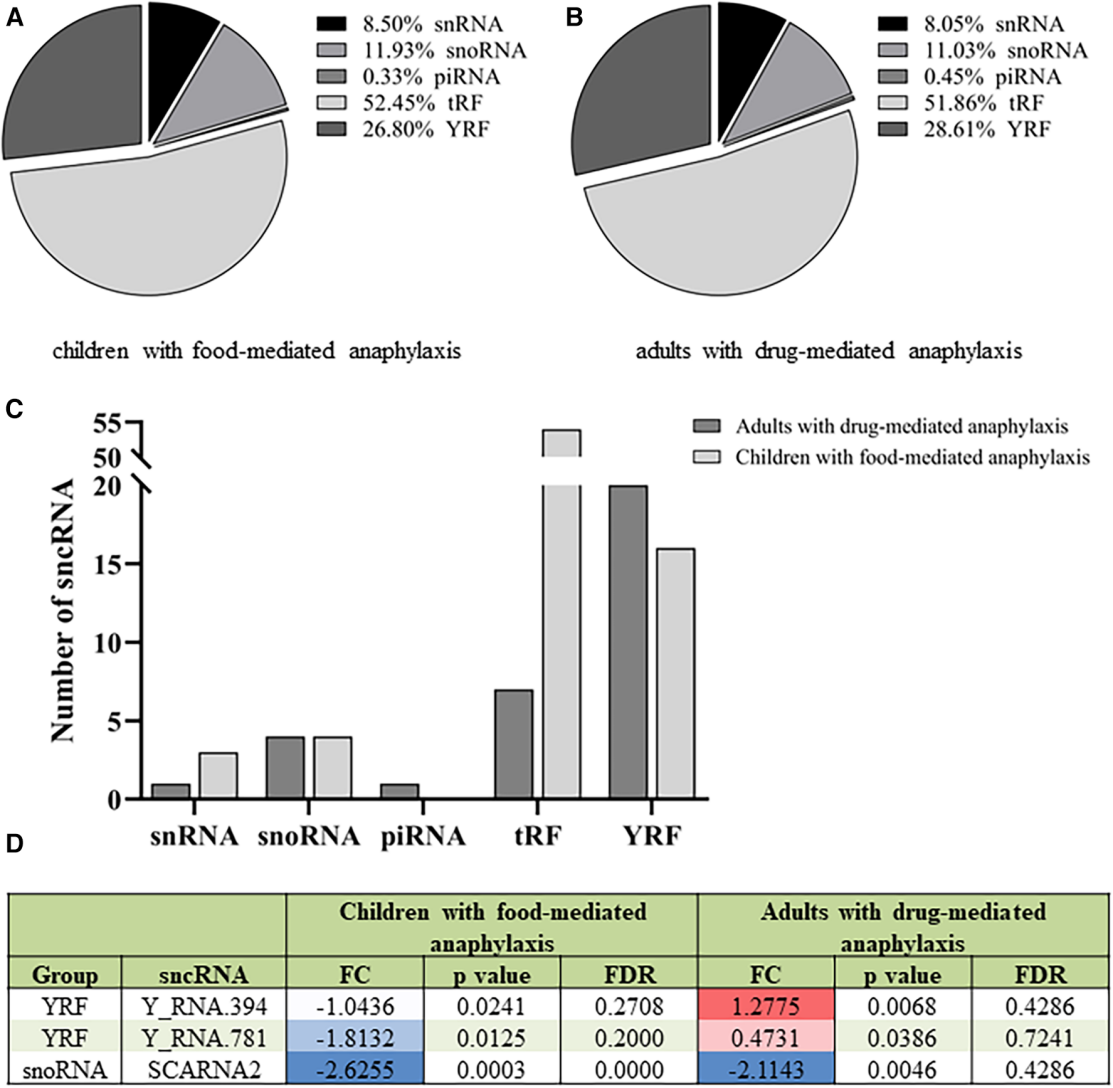
Different sncRNA profiles in children with food-mediated anaphylaxis and adults with drug-mediated anaphylaxis. Profile of the (**A**) 612 sncRNAs identified in the 5 children with food-mediated anaphylaxis and the (**B**) 671 sncRNAs characterized in the 5 adults with drug-mediated anaphylaxis. (**C**) Comparison of the number of statistically significant sncRNAs identified in each cohort according to the specific group. (**D**) Table of the 3 statistically significant sncRNAs common to the 5 children with food-mediated anaphylaxis and the 5 adults with drug-mediated anaphylaxis. FC: fold change (anaphylaxis/control). Positive values indicate an increase (red) during anaphylaxis, while negative values imply a decrease (blue) during the reaction. FDR: false discovery ratio. snRNA, small nuclear RNA; snoRNA, small nucleolar RNA; piRNA, Piwi-interacting RNA; tRF, transference RNA derived fragment; YRF, YRNA derived fragment.

The profile of the 612 sncRNAs found in children with food-mediated anaphylaxis was heterogeneously distributed among the different groups, being 2 piRNAs, 73 snoRNAs, 52 snRNAs, 321 tRFs and 164 YRFs (Figure 2A). However, after statistical analysis, the 80 significant molecules showed a pattern of 4 snoRNAs, 6 snRNAs, 54 tRFs and 16 YRFs (Table 2). On the other hand, the profile of the 671 sncRNAs identified in adults with drug-mediated anaphylaxis was very similar to that of children, being clustered into 3 piRNAs, 74 snoRNAs, 54 snRNAs, 348 tRFs and 192 YRFs (Figure 2B). In turn, the 33 statistically significant molecules described in this cohort were distributed in 1 piRNA, 4 snoRNAs, 1 snRNA, 7 tRFs and 20 YRFs (Table 3).

**Table 2:** List of the 80 statistically significant sncRNAs described in the 5 children with food-mediated anaphylaxis.

Group	sncRNA	FC	*p* value	FDR
YRF	Y_RNA.549	2.1678	0.0031	0.0588
tRF	tRNA174-GlnTTG	1.9402	0.0001	0.0000
tRF	tRNA10-IleAAT	1.8211	0.0236	0.2609
YRF	Y_RNA.630	1.5771	0.0120	0.2000
tRF	tRNA7-LeuCAG	1.3140	0.0327	0.2742
YRF	Y_RNA.10	1.2691	0.0024	0.0588
tRF	tRNA4-ValTAC	1.2560	0.0391	0.2958
YRF	Y_RNA.31	1.2167	0.0160	0.2353
tRF	tRNA96-PseudoCCT	1.2148	0.0325	0.2742
YRF	Y_RNA.479	1.1329	0.0448	0.3117
tRF	tRNA17-ValTAC	1.1120	0.0258	0.2742
tRF	tRNA7-IleGAT	1.0962	0.0434	0.3117
tRF	tRNA32-LysCTT	1.0729	0.0322	0.2742
tRF	tRNA153-IleAAT	1.0664	0.0233	0.2609
snRNA	RNU11	1.0534	0.0460	0.3125
tRF	tRNA3-CysGCA	1.0214	0.0304	0.2742
tRF	tRNA7-LysCTT	1.0084	0.0143	0.2258
tRF	tRNA11-LysCTT	0.8403	0.0164	0.2368
tRF	tRNA20-GluTTC	0.8077	0.0286	0.2742
tRF	tRNA14-LysTTT	0.7987	0.0257	0.2742
tRF	tRNA29-ProAGG	0.7865	0.0104	0.1786
tRF	tRNA9-ProAGG	0.7748	0.0204	0.2609
tRF	tRNA54-LysTTT	0.7547	0.0347	0.2836
tRF	tRNA65-ProAGG	0.7478	0.0159	0.2353
YRF	Y_RNA.147	0.7165	0.0328	0.2769
tRF	tRNA6-ProTGG	0.6936	0.0296	0.2742
tRF	tRNA2-ProAGG	0.6655	0.0383	0.2958
tRF	tRNA30-ProCGG	0.6538	0.0227	0.2609
tRF	tRNA5-GluTTC	0.6029	0.0434	0.3117
tRF	tRNA8-ProTGG	0.5688	0.0429	0.3108
tRF	tRNA40-ValTAC	0.5404	0.0373	0.2899
tRF	tRNA37-ProCGG	0.5192	0.0453	0.3125
snoRNA	SNORA60	0.3772	0.0426	0.3108
YRF	Y_RNA.60	−0.4284	0.0296	0.2742
YRF	Y_RNA.795	−0.5595	0.0407	0.3056
snRNA	RNU1-12P	−0.6824	0.0316	0.2742
tRF	tRNA72-AspGTC	−0.6846	0.0383	0.2899
tRF	tRNA10-AspGTC	−0.6868	0.0341	0.2769
tRF	tRNA81-AspGTC	−0.7753	0.0345	0.2836
YRF	Y_RNA.37	−0.7949	0.0186	0.2500
tRF	tRNA78-AspGTC	−0.8018	0.0168	0.2368
tRF	tRNA69-AspGTC	−0.8018	0.0342	0.2769
tRF	tRNA4-AspGTC	−0.8332	0.0248	0.2708
tRF	tRNA48-AspGTC	−0.8464	0.0229	0.2609
tRF	tRNA38-AspGTC	−0.8547	0.0268	0.2742
tRF	tRNA68-AlaAGC	−0.8914	0.0098	0.1786
tRF	tRNA23-LysTTT	−0.9186	0.0273	0.2742
tRF	tRNA108-AlaAGC	−0.9188	0.0211	0.2609
tRF	tRNA144-AspGTC	−0.9273	0.0161	0.2353
YRF	Y_RNA.565	−0.9330	0.0005	0.0000
snRNA	U1.82	−0.9405	0.0265	0.2742
snoRNA	SNORA63	−0.9651	0.0176	0.2368
tRF	tRNA2-TyrGTA	−0.9682	0.0073	0.1250
tRF	tRNA75-AspGTC	−0.9708	0.0055	0.1250
YRF	Y_RNA.394	−1.0436	0.0241	0.2708
tRF	tRNA45-AspGTC	−1.1078	0.0033	0.0588
tRF	tRNA5-CysGCA	−1.1670	0.0032	0.0588
tRF	tRNA11-LysTTT	−1.1682	0.0462	0.3125
tRF	tRNA31-SerGCT	−1.2347	0.0062	0.1250
tRF	tRNA30-LysCTT	−1.2401	0.0004	0.0000
YRF	Y_RNA.544	−1.2498	0.0012	0.0000
tRF	tRNA17-SupTTA	−1.2711	0.0190	0.2500
snRNA	U2.27	−1.3408	0.0034	0.1111
tRF	tRNA11-PheGAA	−1.3512	0.0002	0.0000
tRF	tRNA16-TyrGTA	−1.3879	0.0015	0.0000
YRF	Y_RNA.349	−1.4396	0.0002	0.0000
tRF	tRNA166-AlaAGC	−1.4581	0.0064	0.1250
tRF	tRNA6-AlaAGC	−1.4876	0.0065	0.1250
YRF	Y_RNA.184	−1.5507	0.0164	0.2368
tRF	tRNA14-TyrGTA	−1.5981	0.0085	0.1600
tRF	tRNA10-SerGCT	−1.6024	0.0007	0.0000
tRF	tRNA10-CysGCA	−1.6164	0.0001	0.0000
tRF	tRNA6-PseudoCTT	−1.6229	0.0093	0.1786
snoRNA	SNORD1B	−1.6910	0.0001	0.0000
tRF	tRNA106-PheGAA	−1.7358	0.0066	0.1250
YRF	Y_RNA.53	−1.7447	0.0019	0.0588
YRF	Y_RNA.781	−1.8132	0.0125	0.2000
snRNA	RNU6−41	−2.1874	0.0295	0.2742
snoRNA	SCARNA2	−2.6255	0.0003	0.0000
snRNA	U2.32	−4.9307	0.0004	0.0000

FC, fold change (anaphylaxis/control). Positive values indicate an increase (red) during anaphylaxis, while negative values imply a decrease (blue) during the reaction. FDR, false discovery ratio.

**Table 3:** List of the 33 statistically significant sncRNAs described in the 5 adults with drug-mediated anaphylaxis.

Group	sncRNA	FC	*p* value	FDR
tRF	tRNA16-GlnTTG	1.6794	0.0056	0.4286
YRF	Y_RNA.625	1.3868	0.0372	0.7241
YRF	Y_RNA.150	1.3130	0.0229	0.6842
YRF	Y_RNA.295	1.3074	0.0095	0.5556
YRF	Y_RNA.201	1.2777	0.0294	0.7241
YRF	Y_RNA.394	1.2775	0.0068	0.4286
YRF	Y_RNA.761	1.2393	0.0063	0.4286
YRF	Y_RNA.445	1.2116	0.0173	0.6250
tRF	tRNA1-PseudoCCC	1.2076	0.0382	0.7241
tRF	tRNA140-LeuCAA	1.1483	0.0167	0.6250
YRF	Y_RNA.30	1.0723	0.0319	0.7241
tRF	tRNA3-ArgTCT	1.0497	0.0089	0.5000
tRF	tRNA33-PseudoAGG	0.9515	0.0014	0.0000
YRF	Y_RNA.288	0.9103	0.0028	0.4286
tRF	tRNA106-HisGTG	0.8983	0.0395	0.7333
YRF	Y_RNA.122	0.8824	0.0475	0.7879
YRF	Y_RNA.700	0.8308	0.0375	0.7241
YRF	Y_RNA.118	0.7969	0.0147	0.6250
YRF	Y_RNA.376	0.7561	0.0189	0.6250
YRF	Y_RNA.413	0.7408	0.0384	0.7241
YRF	Y_RNA.778	0.6528	0.0317	0.7241
YRF	Y_RNA.623	0.5882	0.0198	0.6250
YRF	Y_RNA.781	0.4731	0.0386	0.7241
snoRNA	SNORA3	0.4617	0.0440	0.7500
snoRNA	SNORD41	−0.7450	0.0188	0.6250
snRNA	RNU1-13P	−0.8545	0.0362	0.7241
tRF	tRNA43-SerGCT	−0.9398	0.0422	0.7419
YRF	Y_RNA.605	−0.9642	0.0241	0.6842
snoRNA	SNORD50A	−0.9770	0.0177	0.6250
piRNA	uc011ley.2	−1.1601	0.0281	0.7241
YRF	Y_RNA.49	−1.7268	0.0041	0.4286
YRF	Y_RNA.90	−1.8527	0.0241	0.6842
snoRNA	SCARNA2	−2.1143	0.0046	0.4286

FC, fold change (anaphylaxis/control). Positive values indicate an increase (red) during anaphylaxis, while negative values imply a decrease (blue) during the reaction. FDR, false discovery ratio.

When comparing the sncRNAs profile between both populations, a higher number of significant snRNAs and tRFs were observed in children with food-mediated anaphylaxis. Instead, the only significant piRNA and higher number of YRFs were identified in adults with drug-mediated anaphylaxis (Figure 2C). Moreover, only 3 sncRNAs were common between both cohorts studied, 2 YRFs and 1 snoRNA (Figure 2D).

### Circulating levels of small non-coding RNAs are modulated in anaphylaxis

**Figure 3:**
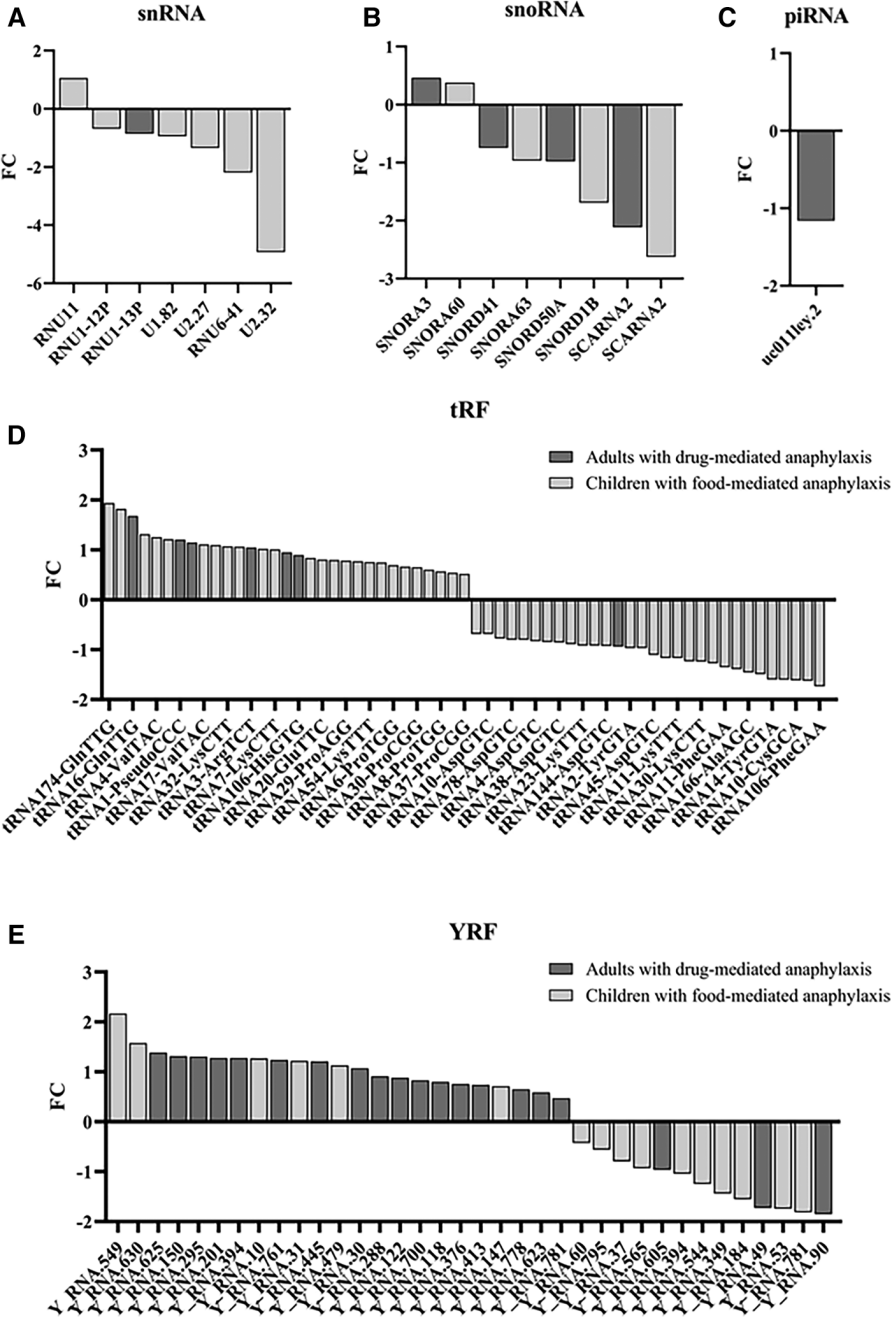
Modulation of the different sncRNA groups during anaphylaxis. Graphical distribution according to their fold change (FC; anaphylaxis/control) of the (**A**) 7 snRNAs, (**B**) 8 snoRNAs, (**C**) 1 piRNA, (**D**) 61 tRFs and (**E**) 36 YRFs found statistically significant in patients with anaphylaxis. snRNA, small nuclear RNA; snoRNA, small nucleolar RNA; piRNA, Piwi-interacting RNA; tRF, transference RNA derived fragment; YRF, YRNA derived fragment.

From all the significant sncRNA, the individual analysis of the specific groups revealed that most of the snRNAs and snoRNAs were decreased during anaphylaxis compared to control samples (Figures 3A,B). Similarly, the only piRNA identified diminished during the reaction (Figure 3C). On the other hand, in relation to tRFs, no differences were observed between the number of molecules that increased during the reaction compared to those that decreased. However, when comparing both cohorts, most of the significantly identified tRFs in adults with drug-mediated anaphylaxis were elevated during the reaction (Figure 3D). In turn, the majority of YRFs increased during anaphylaxis compared to control samples, especially in adults with drug-mediated reactions (Figure 3E).

## Discussion

The life-threatening nature of anaphylaxis fosters a growing interest in the improvement of its clinical management, as well as in the finding of new molecular markers that allows an accurate diagnosis of this pathological event. However, for this purpose, it is necessary to simultaneously increase the molecular knowledge underlying the clinical reaction (13). Precisely, circulating serum sncRNAs are emerging agents that have been described as non-invasive biomarkers and as messengers in the pathophysiology of different diseases (25, 26). Therefore, in this study, we have characterized the profile of other sncRNAs beyond miRNAs in patients with anaphylaxis.

A limitation of “-omics” technologies is the small number of samples used for the analysis, normally due to the high costs of execution. Employing a reduced group of patients to study a very heterogeneous reaction such as anaphylaxis makes it difficult to select a representative set of the population. Therefore, for the development of NGS, individuals with a specific age range and trigger were chosen. Specifically, the study was conducted in children with food-mediated anaphylaxis and adults with drug-mediated anaphylaxis, because they represent the main triggers for each age group (2, 3). In turn, the prevalence of symptoms varied according to age, with respiratory manifestations being more common in children, while cardiovascular and nervous manifestations were more frequent in adults, as previously described (4, 27). However, it could be attributed to the major number of severe reactions included in the adult cohort. Similarly occurs with serum tryptase levels, where the increase is higher in adults with drug-mediated anaphylaxis than in children with food-mediated anaphylaxis. Precisely, it has been described that reactions caused by drugs show higher levels of this biomarker. Nevertheless, the increase of tryptase could also be influenced by differences in the severity of the reaction between both groups (6, 7).

In the allergy field, most of the research has focused special attention elucidating the role of miRNA and lncRNA, particularly those carried by extracellular vesicles (EVs) (28–32). Specifically, differential profiles of miRNAs in children with food-mediated anaphylaxis and adults with drug-mediated anaphylaxis have already been described by our group (20, 21). However, the possible involvement of the other sncRNA is practically unexplored. During the last decades, knowledge of these molecules has expanded and interest in their study has increased (14, 15). Consequently, we set out to characterize for the first time the profiles of other sncRNAs, beyond miRNAs, in patients with anaphylaxis. Our data demonstrated similar identification profiles between adults with drug-mediated anaphylaxis and children with food-mediated anaphylaxis. Nevertheless, after statistical analysis, a high number of molecules were seen in the pediatric population. In addition, the profiles became almost completely different, with only 3 common sncRNAs between them. Divergences in patterns would be attributed to variations associated with triggers and their underlying molecular mechanisms. Indeed, food-triggered anaphylactic reactions are characterized by being mainly IgE-mediated, whereas drug-induced reactions could be associated with both IgE and non-IgE mechanisms (33). For instance, NSAIDs, the most frequent allergen in the adult population, leads mainly non-IgE reactions (34). Therefore, the lower number of significant sncRNAs observed in the adult group could be due to less homogeneity among patients, being a limitation of our study. However, differences between the two cohort profiles could also indicate that specific sncRNA mechanisms exists. In particular, the two YRFs (Y_RNA.394 and Y_RNA.781) found common in both populations showed different behavior in each cohort, suggesting its possible utility as markers to discriminate different endotypes of the reaction. Instead, the common snoRNA (SCARNA2) was downregulated in both cohorts, appearing as a biomarker of anaphylaxis, independently of the age differences between both populations and/or the underlying molecular mechanism. Nevertheless, future studies that validate findings on a larger number of samples are needed to determine the relationship of these molecules with anaphylactic endotypes.

Among the different populations of sncRNAs identified by NGS, tRFs were the group with highest variations in children with food-mediated anaphylaxis. These molecules have become central for several studies because of their similarity to miRNAs. Precisely, tRFs may be involved in Ago-mediated translation repression. Conversely, they may also participate in other cellular activities including cell proliferation, transposon silencing and epigenetic inheritance (25, 35). In addition, tRFs have been proposed as biomarkers for diseases such as cancer and osteoporosis (36, 37). Currently, evidence between them and human pathologies remain descriptive. However, tRFs have been found to be upregulated under oxidative stress conditions (25, 35). Therefore, tRFs variations observed in patients with anaphylaxis could be due to their relationship with the oxidative stress concomitant to this pathological event (38). Particularly in the case of adults with drug-mediated anaphylaxis, where fewer differential molecules were observed, but most of them increased during the reaction. On the other hand, YRFs were the group with the highest variations in the adult cohort. These transcripts are fragments derived from YRNAs molecules which have been observed to be overexpressed in allergic patients (39). However, most studies involving YRFs have only been conducted in the cancer field, where they have been proposed as diagnostic markers. Unfortunately, their molecular function has not been fully defined yet (40, 41). Nevertheless, one study showed that YRFs regulate cell death and inflammation in monocytes/macrophages (42), which are relevant cells for the development of anaphylaxis, especially in non-IgE-mediated reactions (12, 13). Therefore, the increase of YRFs could be related to a modulation of monocyte and macrophage activation in anaphylaxis. In turn, since the number of identified snRNAs and snoRNAs was similar in both studied cohorts, the statistically significant snRNAs were more abundant in children with food-mediated anaphylaxis. Moreover, both groups of sncRNAs showed a homogeneous behavior, decreasing their levels during anaphylaxis. Contrary, the only study of other sncRNAs beyond miRNAs conducted in anaphylaxis identified an increase of snoRNAs during the reaction (17). Unfortunately, none of those was identified in our studies. Likely, the recruiting timing of the samples used to carry out the analysis is responsible of these differences. Finally, piRNAs were almost absent compared to the rest of the sncRNAs and practically none of them were statistically different between the two conditions evaluated.

## Conclusion

In this study, we have identified a specific profile of 80 sncRNAs (4 snoRNAs, 6 snRNAs, 54 tRFs and 16 YRFs) in children with food-mediated anaphylaxis and 33 sncRNAs (1 piRNA, 4 snoRNAs, 1 snRNAs, 7 tRFs and 20 YRFs) in adults with drug-mediated anaphylaxis. Nevertheless, among them, only three molecules (Y_RNA.394, Y_RNA.781 and SCARNA2) were common to both analyses.

Although preliminary, our findings suggest the scientific potential of sncRNAs and will serve as a basis for further studies, possibly leading to new clinical strategies. However, it is still necessary to validate their potential diagnostic utility, as well as to determine their possible involvement in the different molecular mechanisms underlying anaphylaxis.

## Data Availability

The data presented in the study are deposited in the GEO-NCBI repository, accession number GSE245653.
