# Supplementary material for: Circulating serum profile of small non-coding RNAs in patients with anaphylaxis beyond microRNAs
Source: Front Allergy. 2024 Feb 7;5:1307880. doi: 10.3389/falgy.2024.1307880 (PMC10879566; doi:10.3389/falgy.2024.1307880)
Supplement: Supplementary file 1 [file Table1.docx]

**SUPPLEMENTARY MATERIAL**

**SUPPLEMENTARY TABLE 1**

| **Group** | **sncRNA** | **FC** | **p value** | **FDR** |
| --- | --- | --- | --- | --- |
| snRNA | RNU1-10P | 0.0353 | 0.8493 | 0.8677 |
| snRNA | RNU1-11P | 0.1620 | 0.6594 | 0.8241 |
| snRNA | RNU11 | 1.0534 | 0.0460 | 0.3125 |
| snRNA | RNU1-1 | 0.1033 | 0.6532 | 0.8210 |
| snRNA | RNU1-12P | -0.6824 | 0.0316 | 0.2742 |
| snRNA | RNU1-13P | -0.7247 | 0.1177 | 0.4658 |
| snRNA | RNU12 | 0.2846 | 0.3760 | 0.6667 |
| snRNA | RNU1-2 | -0.1599 | 0.5699 | 0.7716 |
| snRNA | RNU1-22P | 0.7201 | 0.3372 | 0.6608 |
| snRNA | RNU1-3 | 0.6796 | 0.0720 | 0.3774 |
| snRNA | RNU1-4 | 0.1139 | 0.6741 | 0.8320 |
| snRNA | RNU1-5 | -0.3936 | 0.2280 | 0.5749 |
| snRNA | RNU1-6 | 0.6091 | 0.2417 | 0.5794 |
| snRNA | RNU1-7 | 0.0990 | 0.7191 | 0.8416 |
| snRNA | RNU1-8 | -0.0101 | 0.8606 | 0.8693 |
| snRNA | RNU1-9 | 0.1645 | 0.5776 | 0.7747 |
| snRNA | RNU2-2 | -0.4710 | 0.2148 | 0.5749 |
| snRNA | RNU2-3P | 0.0177 | 0.8219 | 0.8623 |
| snRNA | RNU2-4P | -0.5716 | 0.3097 | 0.6395 |
| snRNA | RNU2-5P | -0.2195 | 0.5390 | 0.7633 |
| snRNA | RNU2-6P | -0.3429 | 0.2549 | 0.5932 |
| snRNA | RNU2-7P | -0.2139 | 0.2781 | 0.6079 |
| snRNA | RNU5A-1 | 0.3474 | 0.6305 | 0.8118 |
| snRNA | RNU5B-1 | -0.2620 | 0.6813 | 0.8330 |
| snRNA | RNU5E-1 | -0.7319 | 0.1715 | 0.5455 |
| snRNA | RNU6-41 | -2.1874 | 0.0295 | 0.2742 |
| snoRNA | SCARNA15 | 0.0903 | 0.6598 | 0.8241 |
| snoRNA | SCARNA16 | 1.5747 | 0.1096 | 0.4653 |
| snoRNA | SCARNA17 | -0.5340 | 0.3000 | 0.6293 |
| snoRNA | SCARNA18.2 | 0.5878 | 0.4125 | 0.6923 |
| snoRNA | SCARNA2 | -2.6255 | 0.0003 | 0.0000 |
| snoRNA | SCARNA3 | 0.6255 | 0.1693 | 0.5405 |
| snoRNA | SCARNA5 | -0.9286 | 0.2716 | 0.6036 |
| snoRNA | SCARNA7 | -0.0403 | 1.0000 | 0.8693 |
| snoRNA | SCARNA8 | 0.2805 | 0.4923 | 0.7506 |
| snoRNA | SNORA3 | 0.3699 | 0.4911 | 0.7506 |
| snoRNA | SNORA54 | -1.0241 | 0.1328 | 0.4940 |
| snoRNA | SNORA60 | 0.3772 | 0.0426 | 0.3108 |
| snoRNA | SNORA63 | -0.9651 | 0.0176 | 0.2368 |
| snoRNA | SNORA64 | -0.9626 | 0.3871 | 0.6667 |
| snoRNA | SNORA73B | 1.2671 | 0.2192 | 0.5749 |
| snoRNA | SNORD100 | -0.3192 | 0.5793 | 0.7747 |
| snoRNA | SNORD102 | 1.3463 | 0.1955 | 0.5747 |
| snoRNA | SNORD104 | 0.1574 | 0.7462 | 0.8416 |
| snoRNA | SNORD110 | -0.9005 | 0.2497 | 0.5869 |
| snoRNA | SNORD118 | -0.3496 | 0.3701 | 0.6667 |
| snoRNA | SNORD119 | 0.8391 | 0.3645 | 0.6667 |
| snoRNA | SNORD15A | 0.1695 | 0.6841 | 0.8330 |
| snoRNA | SNORD1B | -1.6910 | 0.0001 | 0.0000 |
| snoRNA | SNORD2.2 | -1.2957 | 0.2031 | 0.5747 |
| snoRNA | SNORD20 | 1.5554 | 0.1895 | 0.5747 |
| snoRNA | SNORD21 | -0.4810 | 0.4191 | 0.6979 |
| snoRNA | SNORD22 | 0.8659 | 0.2990 | 0.6293 |
| snoRNA | SNORD24 | 1.0544 | 0.2330 | 0.5749 |
| snoRNA | SNORD26 | -0.0919 | 0.7959 | 0.8482 |
| snoRNA | SNORD30 | -1.4520 | 0.2083 | 0.5747 |
| snoRNA | SNORD31 | -0.1267 | 0.7716 | 0.8466 |
| snoRNA | SNORD32A | 0.0163 | 0.8665 | 0.8693 |
| snoRNA | SNORD33 | 0.3235 | 0.3746 | 0.6667 |
| snoRNA | SNORD38A | -1.4387 | 0.1508 | 0.5000 |
| snoRNA | SNORD38B | -0.2662 | 0.6466 | 0.8191 |
| snoRNA | SNORD3A | -0.3760 | 0.3863 | 0.6667 |
| snoRNA | SNORD3B-1 | -0.1945 | 0.5907 | 0.7821 |
| snoRNA | SNORD3B-2 | -0.4135 | 0.2948 | 0.6293 |
| snoRNA | SNORD3C | -0.3354 | 0.4095 | 0.6923 |
| snoRNA | SNORD3D | -0.3819 | 0.3423 | 0.6614 |
| snoRNA | SNORD41 | 0.9327 | 0.3511 | 0.6667 |
| snoRNA | SNORD45A | -1.9169 | 0.0837 | 0.3967 |
| snoRNA | SNORD47 | -1.1485 | 0.1801 | 0.5625 |
| snoRNA | SNORD48 | -1.6976 | 0.1402 | 0.4940 |
| snoRNA | SNORD50A | 0.2595 | 1.0000 | 0.8693 |
| snoRNA | SNORD50B | -0.5022 | 0.5388 | 0.7633 |
| snoRNA | SNORD51 | -1.0102 | 0.1355 | 0.4940 |
| snoRNA | SNORD52 | -0.9403 | 1.0000 | 0.8693 |
| snoRNA | SNORD61 | -0.2060 | 1.0000 | 0.8693 |
| snoRNA | SNORD6 | 0.6494 | 0.2822 | 0.6165 |
| snoRNA | SNORD62A | 0.8210 | 0.2062 | 0.5747 |
| snoRNA | SNORD62B | -0.0111 | 0.8638 | 0.8693 |
| snoRNA | SNORD63 | -0.2752 | 0.7248 | 0.8416 |
| snoRNA | SNORD66 | -0.7843 | 0.2251 | 0.5749 |
| snoRNA | SNORD68 | 1.0027 | 0.2447 | 0.5837 |
| snoRNA | SNORD69 | 0.1886 | 0.7454 | 0.8416 |
| snoRNA | SNORD71 | -0.0293 | 0.8501 | 0.8677 |
| snoRNA | SNORD74 | 0.6636 | 0.4203 | 0.6979 |
| snoRNA | SNORD80 | 0.2991 | 0.7418 | 0.8416 |
| snoRNA | SNORD81 | -0.8946 | 0.2970 | 0.6293 |
| snoRNA | SNORD82 | -1.8824 | 0.2232 | 0.5749 |
| snoRNA | SNORD83A | 0.3852 | 0.5368 | 0.7633 |
| snoRNA | SNORD83B | 0.6298 | 0.4464 | 0.7158 |
| snoRNA | SNORD84-203 | 0.6009 | 0.4311 | 0.7013 |
| snoRNA | SNORD85 | 1.2152 | 0.3285 | 0.6515 |
| snoRNA | SNORD89 | -0.1456 | 0.5008 | 0.7568 |
| snoRNA | SNORD90 | 0.6146 | 0.5390 | 0.7633 |
| snoRNA | SNORD94 | -0.0852 | 0.6415 | 0.8146 |
| snoRNA | SNORD95 | -0.1964 | 0.7219 | 0.8416 |
| snoRNA | SNORD96A | -1.3693 | 0.0894 | 0.4109 |
| snoRNA | SNORD97 | 0.3467 | 0.4694 | 0.7385 |
| snoRNA | SNORD99 | 0.2487 | 0.6690 | 0.8293 |
| snoRNA | snoU2-30.1 | -0.9202 | 0.2059 | 0.5747 |
| tRF | tRNA100-LeuCAA | 0.6253 | 0.1118 | 0.4653 |
| tRF | tRNA101-AlaAGC | 0.2529 | 0.4866 | 0.7494 |
| tRF | tRNA102-AlaAGC | -0.0850 | 0.7324 | 0.8416 |
| tRF | tRNA103-AsnGTT | 0.0951 | 0.7747 | 0.8466 |
| tRF | tRNA104-AlaTGC | -1.0629 | 0.1360 | 0.4940 |
| tRF | tRNA106-HisGTG | 0.5096 | 0.3716 | 0.6667 |
| tRF | tRNA106-PheGAA | -1.7358 | 0.0066 | 0.1250 |
| tRF | tRNA107-AsnGTT | 0.1833 | 0.6236 | 0.8085 |
| tRF | tRNA108-AlaAGC | -0.9188 | 0.0211 | 0.2609 |
| tRF | tRNA108-AsnGTT | 0.2405 | 0.4517 | 0.7199 |
| tRF | tRNA10-AlaCGC | 0.1013 | 0.5684 | 0.7716 |
| tRF | tRNA10-AspGTC | -0.6868 | 0.0341 | 0.2769 |
| tRF | tRNA10-CysGCA | -1.6164 | 0.0001 | 0.0000 |
| tRF | tRNA10-GlyTCC | -0.2090 | 0.4704 | 0.7385 |
| tRF | tRNA10-IleAAT | 1.8211 | 0.0236 | 0.2609 |
| tRF | tRNA10-LysCTT | 0.4477 | 0.1439 | 0.4941 |
| tRF | tRNA10-MetCAT | -0.1773 | 0.5306 | 0.7633 |
| tRF | tRNA10-PseudoCTC | 1.0166 | 0.0807 | 0.3967 |
| tRF | tRNA10-SerGCT | -1.6024 | 0.0007 | 0.0000 |
| tRF | tRNA10-ValCAC | 0.0972 | 0.5293 | 0.7633 |
| tRF | tRNA110-AlaTGC | -0.3672 | 0.5998 | 0.7935 |
| tRF | tRNA111-HisGTG | 0.7326 | 0.0521 | 0.3333 |
| tRF | tRNA113-AlaTGC | -0.3792 | 0.4421 | 0.7124 |
| tRF | tRNA115-ValAAC | -0.3324 | 0.5702 | 0.7716 |
| tRF | tRNA116-GluCTC | 0.0732 | 0.7073 | 0.8416 |
| tRF | tRNA117-GlyTCC | -0.2158 | 0.6720 | 0.8316 |
| tRF | tRNA118-HisGTG | 0.4867 | 0.1236 | 0.4770 |
| tRF | tRNA119-AlaCGC | 0.3737 | 0.3580 | 0.6667 |
| tRF | tRNA119-LysCTT | 0.5977 | 0.0564 | 0.3444 |
| tRF | tRNA11-ArgACG | 1.0163 | 0.1248 | 0.4770 |
| tRF | tRNA11-GluTTC | 0.6715 | 0.0719 | 0.3774 |
| tRF | tRNA11-IleAAT | 0.3715 | 0.4367 | 0.7101 |
| tRF | tRNA11-LysCTT | 0.8403 | 0.0164 | 0.2368 |
| tRF | tRNA11-LysTTT | -1.1682 | 0.0462 | 0.3125 |
| tRF | tRNA11-PheGAA | -1.3512 | 0.0002 | 0.0000 |
| tRF | tRNA11-ProAGG | 0.3674 | 0.1000 | 0.4318 |
| tRF | tRNA11-SerAGA | 0.1567 | 0.6233 | 0.8085 |
| tRF | tRNA120-AlaAGC | 0.3277 | 0.2337 | 0.5749 |
| tRF | tRNA123-SerGCT | -0.8738 | 0.1123 | 0.4653 |
| tRF | tRNA126-LeuAAG | 0.3581 | 0.2213 | 0.5749 |
| tRF | tRNA127-CysGCA | -0.1490 | 0.7091 | 0.8416 |
| tRF | tRNA127-ThrTGT | 0.4124 | 0.3834 | 0.6667 |
| tRF | tRNA128-GlyGCC | -0.0299 | 0.7906 | 0.8482 |
| tRF | tRNA128-LysCTT | 0.1116 | 0.7206 | 0.8416 |
| tRF | tRNA129-MetCAT | -0.0972 | 0.7294 | 0.8416 |
| tRF | tRNA12-ArgCCT | 0.6800 | 1.0000 | 0.8693 |
| tRF | tRNA12-AspGTC | -0.6010 | 0.0512 | 0.3333 |
| tRF | tRNA12-ProAGG | 0.7237 | 0.0663 | 0.3725 |
| tRF | tRNA12-ProTGG | 0.4185 | 0.1707 | 0.5430 |
| tRF | tRNA12-TrpCCA | 0.4313 | 0.1771 | 0.5579 |
| tRF | tRNA12-ValAAC | 0.2432 | 0.2654 | 0.6000 |
| tRF | tRNA130-GlnTTG | -0.1734 | 0.6862 | 0.8330 |
| tRF | tRNA131-GlyCCC | 0.2942 | 0.2963 | 0.6293 |
| tRF | tRNA132-ValAAC | 0.2311 | 0.2331 | 0.5749 |
| tRF | tRNA133-GlyCCC | -0.0114 | 0.8350 | 0.8659 |
| tRF | tRNA133-ValCAC | 0.7014 | 0.0906 | 0.4109 |
| tRF | tRNA134-GluTTC | 0.3450 | 0.1746 | 0.5532 |
| tRF | tRNA134-LeuTAA | -0.3627 | 0.5104 | 0.7633 |
| tRF | tRNA136-ValAAC | 0.1765 | 0.4119 | 0.6923 |
| tRF | tRNA137-Pseudo??? | 0.2721 | 0.3786 | 0.6667 |
| tRF | tRNA137-SerCGA | 0.3110 | 0.3620 | 0.6667 |
| tRF | tRNA138-ArgACG | -0.8687 | 0.1151 | 0.4653 |
| tRF | tRNA139-ValAAC | 0.0311 | 0.7672 | 0.8466 |
| tRF | tRNA13-AlaCGC | 0.6005 | 0.2273 | 0.5749 |
| tRF | tRNA13-AlaTGC | -0.0629 | 0.7236 | 0.8416 |
| tRF | tRNA13-GlyCCC | 0.1869 | 0.5125 | 0.7633 |
| tRF | tRNA13-LysCTT | 0.4940 | 0.1107 | 0.4653 |
| tRF | tRNA13-ValCAC | 0.3741 | 0.3276 | 0.6515 |
| tRF | tRNA141-LeuCAA | 0.2591 | 0.5184 | 0.7633 |
| tRF | tRNA142-MetCAT | -0.0411 | 0.8303 | 0.8659 |
| tRF | tRNA143-LysTTT | 0.5227 | 0.1835 | 0.5641 |
| tRF | tRNA144-AspGTC | -0.9273 | 0.0161 | 0.2353 |
| tRF | tRNA145-SerAGA | 0.1219 | 0.6055 | 0.7948 |
| tRF | tRNA146-GlnCTG | 0.3669 | 0.2655 | 0.6000 |
| tRF | tRNA147-SerAGA | 0.0582 | 0.7534 | 0.8440 |
| tRF | tRNA148-SerTGA | 0.0857 | 0.7187 | 0.8416 |
| tRF | tRNA149-LysTTT | -0.3302 | 0.3797 | 0.6667 |
| tRF | tRNA14-LysTTT | 0.7987 | 0.0257 | 0.2742 |
| tRF | tRNA14-ProTGG | 0.1296 | 0.5497 | 0.7691 |
| tRF | tRNA14-ThrCGT | -0.7890 | 0.1167 | 0.4653 |
| tRF | tRNA14-TyrGTA | -1.5981 | 0.0085 | 0.1600 |
| tRF | tRNA150-MetCAT | -0.2960 | 0.4988 | 0.7550 |
| tRF | tRNA151-ThrCGT | -0.2598 | 0.6066 | 0.7948 |
| tRF | tRNA152-ValCAC | 0.5892 | 0.2569 | 0.5932 |
| tRF | tRNA153-IleAAT | 1.0664 | 0.0233 | 0.2609 |
| tRF | tRNA154-IleAAT | 0.4533 | 0.4066 | 0.6908 |
| tRF | tRNA157-ValCAC | 0.4196 | 0.2053 | 0.5747 |
| tRF | tRNA158-IleAAT | 0.7767 | 0.1978 | 0.5747 |
| tRF | tRNA15-CysGCA | -0.1609 | 0.5624 | 0.7691 |
| tRF | tRNA15-ThrCGT | -0.0871 | 0.7796 | 0.8466 |
| tRF | tRNA15-TyrGTA | -0.5943 | 0.1570 | 0.5138 |
| tRF | tRNA15-ValAAC | -0.2401 | 0.2672 | 0.6000 |
| tRF | tRNA163-IleAAT | -0.3089 | 0.6860 | 0.8330 |
| tRF | tRNA165-IleAAT | 0.6399 | 0.2743 | 0.6051 |
| tRF | tRNA166-AlaAGC | -1.4581 | 0.0064 | 0.1250 |
| tRF | tRNA169-MetCAT | -0.0284 | 0.8239 | 0.8625 |
| tRF | tRNA16-CysGCA | -0.1202 | 0.7493 | 0.8419 |
| tRF | tRNA16-GlnTTG | -0.5113 | 0.0829 | 0.3967 |
| tRF | tRNA16-HisGTG | 0.4294 | 0.2295 | 0.5749 |
| tRF | tRNA16-LeuAAG | 0.1398 | 0.5726 | 0.7721 |
| tRF | tRNA16-TyrGTA | -1.3879 | 0.0015 | 0.0000 |
| tRF | tRNA16-ValTAC | -0.2615 | 0.3086 | 0.6395 |
| tRF | tRNA171-MetCAT | 0.0360 | 0.8381 | 0.8659 |
| tRF | tRNA172-SerTGA | 0.3522 | 0.3568 | 0.6667 |
| tRF | tRNA173-GlnTTG | -0.2086 | 0.4113 | 0.6923 |
| tRF | tRNA174-GlnTTG | 1.9402 | 0.0001 | 0.0000 |
| tRF | tRNA175-SerGCT | 0.4922 | 0.2219 | 0.5749 |
| tRF | tRNA17-LeuCAG | 0.1453 | 0.6999 | 0.8356 |
| tRF | tRNA17-PseudoTTC | 0.0780 | 0.6256 | 0.8089 |
| tRF | tRNA17-SupTTA | -1.2711 | 0.0190 | 0.2500 |
| tRF | tRNA17-TyrGTA | -1.9469 | 0.1024 | 0.4436 |
| tRF | tRNA17-ValTAC | 1.1120 | 0.0258 | 0.2742 |
| tRF | tRNA18-ArgCCT | -0.4802 | 0.5423 | 0.7639 |
| tRF | tRNA18-GlyGCC | 0.2456 | 0.3487 | 0.6667 |
| tRF | tRNA18-ValCAC | -0.0384 | 0.7485 | 0.8416 |
| tRF | tRNA19-ArgTCG | 0.1059 | 0.7815 | 0.8466 |
| tRF | tRNA19-GlyGCC | 0.2319 | 0.3288 | 0.6515 |
| tRF | tRNA19-LeuAAG | 0.5213 | 0.2272 | 0.5749 |
| tRF | tRNA19-TyrGTA | -0.6095 | 0.4280 | 0.6979 |
| tRF | tRNA1-AsnGTT | 0.9463 | 0.0938 | 0.4109 |
| tRF | tRNA1-GlnCTG | 0.3119 | 0.4127 | 0.6923 |
| tRF | tRNA1-HisGTG | 0.6413 | 0.0926 | 0.4109 |
| tRF | tRNA1-LeuAAG | 0.5401 | 0.2179 | 0.5749 |
| tRF | tRNA1-PheGAA | -1.1677 | 0.0578 | 0.3516 |
| tRF | tRNA1-SeCTCA | -0.1785 | 0.7216 | 0.8416 |
| tRF | tRNA20-GluTTC | 0.8077 | 0.0286 | 0.2742 |
| tRF | tRNA20-MetCAT | 0.0742 | 0.7954 | 0.8482 |
| tRF | tRNA20-ThrTGT | -0.0751 | 0.7970 | 0.8482 |
| tRF | tRNA21-ArgCCT | 0.7490 | 0.5383 | 0.7633 |
| tRF | tRNA21-HisGTG | 0.6325 | 0.0548 | 0.3371 |
| tRF | tRNA21-ThrTGT | -0.3427 | 0.5613 | 0.7691 |
| tRF | tRNA22-AspGTC | -0.0081 | 0.8617 | 0.8693 |
| tRF | tRNA22-MetCAT | -0.0056 | 0.8670 | 0.8693 |
| tRF | tRNA22-ProAGG | 0.6462 | 0.0543 | 0.3371 |
| tRF | tRNA23-ArgCCG | 0.3852 | 0.3265 | 0.6515 |
| tRF | tRNA23-LysTTT | -0.9186 | 0.0273 | 0.2742 |
| tRF | tRNA23-ProAGG | 0.3908 | 0.2038 | 0.5747 |
| tRF | tRNA24-GlyGCC | 0.0558 | 0.7397 | 0.8416 |
| tRF | tRNA24-LysCTT | -0.0668 | 0.8117 | 0.8549 |
| tRF | tRNA25-GlyGCC | -0.0218 | 0.8193 | 0.8603 |
| tRF | tRNA25-PseudoCTC | 0.3853 | 0.1948 | 0.5747 |
| tRF | tRNA26-AsnGTT | 0.1473 | 0.6911 | 0.8340 |
| tRF | tRNA26-CysGCA | 0.1960 | 0.6790 | 0.8330 |
| tRF | tRNA26-LeuCAG | -0.1828 | 0.6189 | 0.8084 |
| tRF | tRNA27-CysGCA | -0.2833 | 0.4226 | 0.6979 |
| tRF | tRNA27-GlyCCC | -0.7997 | 0.0634 | 0.3684 |
| tRF | tRNA27-LeuTAG | -0.2266 | 0.2935 | 0.6293 |
| tRF | tRNA28-CysGCA | -0.4892 | 0.2056 | 0.5747 |
| tRF | tRNA28-IleAAT | 0.5694 | 0.2698 | 0.6022 |
| tRF | tRNA28-ProTGG | 0.3936 | 0.2076 | 0.5747 |
| tRF | tRNA29-ProAGG | 0.7865 | 0.0104 | 0.1786 |
| tRF | tRNA2-ArgCCT | 0.4261 | 0.3788 | 0.6667 |
| tRF | tRNA2-GlyCCC | 0.6830 | 0.0511 | 0.3333 |
| tRF | tRNA2-GlyGCC | 0.1127 | 0.6963 | 0.8356 |
| tRF | tRNA2-GlyTCC | 0.2937 | 0.2232 | 0.5749 |
| tRF | tRNA2-LeuTAG | -0.2049 | 0.5696 | 0.7716 |
| tRF | tRNA2-LysCTT | 0.4516 | 0.2006 | 0.5747 |
| tRF | tRNA2-LysTTT | 0.5599 | 0.1206 | 0.4698 |
| tRF | tRNA2-MetCAT | -0.1581 | 0.6862 | 0.8330 |
| tRF | tRNA2-ProAGG | 0.6655 | 0.0383 | 0.2958 |
| tRF | tRNA2-PseudoCTC | 1.0197 | 0.0678 | 0.3725 |
| tRF | tRNA2-SerCGA | 0.0695 | 0.6864 | 0.8330 |
| tRF | tRNA2-SerTGA | -0.1088 | 0.5551 | 0.7691 |
| tRF | tRNA2-TyrGTA | -0.9682 | 0.0073 | 0.1250 |
| tRF | tRNA2-ValAAC | 0.1458 | 0.4825 | 0.7481 |
| tRF | tRNA2-ValCAC | 0.3379 | 0.2466 | 0.5837 |
| tRF | tRNA30-CysGCA | -0.4575 | 0.3841 | 0.6667 |
| tRF | tRNA30-LysCTT | -1.2401 | 0.0004 | 0.0000 |
| tRF | tRNA30-ProCGG | 0.6538 | 0.0227 | 0.2609 |
| tRF | tRNA31-AsnGTT | 0.0843 | 0.7206 | 0.8416 |
| tRF | tRNA31-PseudoTGG | -1.0208 | 0.0693 | 0.3725 |
| tRF | tRNA31-SerGCT | -1.2347 | 0.0062 | 0.1250 |
| tRF | tRNA32-LysCTT | 1.0729 | 0.0322 | 0.2742 |
| tRF | tRNA32-MetCAT | -0.3113 | 0.4762 | 0.7398 |
| tRF | tRNA33-HisGTG | 0.4656 | 0.0859 | 0.3967 |
| tRF | tRNA34-GlyCCC | -0.5975 | 0.1315 | 0.4935 |
| tRF | tRNA34-IleAAT | 0.7002 | 0.1851 | 0.5685 |
| tRF | tRNA34-LeuCAG | 0.5274 | 0.2480 | 0.5837 |
| tRF | tRNA35-GlyGCC | 0.1947 | 0.5605 | 0.7691 |
| tRF | tRNA35-SerAGA | 0.1189 | 0.6817 | 0.8330 |
| tRF | tRNA35-SerCGA | 0.1182 | 0.5320 | 0.7633 |
| tRF | tRNA36-LeuCAG | -0.7297 | 0.1128 | 0.4653 |
| tRF | tRNA37-GlyGCC | 0.1765 | 0.6234 | 0.8085 |
| tRF | tRNA37-ProCGG | 0.5192 | 0.0453 | 0.3125 |
| tRF | tRNA37-ValAAC | 0.5180 | 0.1457 | 0.4942 |
| tRF | tRNA38-AspGTC | -0.8547 | 0.0268 | 0.2742 |
| tRF | tRNA38-IleAAT | -0.4735 | 0.2116 | 0.5747 |
| tRF | tRNA38-LeuCAG | 0.0761 | 0.7541 | 0.8443 |
| tRF | tRNA39-GlyGCC | -0.0449 | 0.7958 | 0.8482 |
| tRF | tRNA39-PseudoAAT | -0.2121 | 0.7952 | 0.8482 |
| tRF | tRNA3-AlaAGC | 0.7438 | 0.1355 | 0.4940 |
| tRF | tRNA3-ArgCCT | -0.2655 | 0.5507 | 0.7691 |
| tRF | tRNA3-ArgTCT | 0.1251 | 0.6531 | 0.8210 |
| tRF | tRNA3-CysGCA | 1.0214 | 0.0304 | 0.2742 |
| tRF | tRNA3-GlnCTG | -0.5633 | 0.3830 | 0.6667 |
| tRF | tRNA3-GluTTC | 0.7476 | 0.1253 | 0.4771 |
| tRF | tRNA3-LeuAAG | 0.3822 | 0.1509 | 0.5000 |
| tRF | tRNA3-ProTGG | -0.1172 | 0.5589 | 0.7691 |
| tRF | tRNA3-PseudoCAC | 0.1416 | 0.7366 | 0.8416 |
| tRF | tRNA40-LeuCAG | -0.0286 | 0.8430 | 0.8668 |
| tRF | tRNA40-ValTAC | 0.5404 | 0.0373 | 0.2899 |
| tRF | tRNA41-GlyGCC | 0.3336 | 0.3688 | 0.6667 |
| tRF | tRNA41-PseudoACT | 0.5490 | 0.3561 | 0.6667 |
| tRF | tRNA41-SerCGA | 0.2238 | 0.3265 | 0.6515 |
| tRF | tRNA42-LeuCAG | 0.0242 | 0.8437 | 0.8668 |
| tRF | tRNA42-LeuTAG | 0.4708 | 0.0530 | 0.3333 |
| tRF | tRNA43-GlyGCC | 0.4146 | 0.1392 | 0.4940 |
| tRF | tRNA43-SerGCT | -0.4162 | 0.3180 | 0.6500 |
| tRF | tRNA44-SerAGA | 0.0340 | 0.8077 | 0.8544 |
| tRF | tRNA45-AspGTC | -1.1078 | 0.0033 | 0.0588 |
| tRF | tRNA45-GlyTCC | -0.1468 | 0.6377 | 0.8121 |
| tRF | tRNA46-SerAGA | -0.1689 | 0.5097 | 0.7633 |
| tRF | tRNA47-AsnGTT | -0.1737 | 0.6368 | 0.8121 |
| tRF | tRNA47-SerAGA | 0.3710 | 0.2714 | 0.6022 |
| tRF | tRNA48-AspGTC | -0.8464 | 0.0229 | 0.2609 |
| tRF | tRNA49-GlnCTG | 0.3883 | 0.3190 | 0.6500 |
| tRF | tRNA4-ArgTCG | -0.8262 | 0.1406 | 0.4940 |
| tRF | tRNA4-ArgTCT | -0.1169 | 0.6539 | 0.8210 |
| tRF | tRNA4-AsnGTT | 0.5204 | 0.3651 | 0.6667 |
| tRF | tRNA4-AspGTC | -0.8332 | 0.0248 | 0.2708 |
| tRF | tRNA4-GlyCCC | 0.1202 | 0.5310 | 0.7633 |
| tRF | tRNA4-LeuTAA | -0.4669 | 0.5380 | 0.7633 |
| tRF | tRNA4-LysCTT | 0.4597 | 0.3150 | 0.6481 |
| tRF | tRNA4-ProAGG | 1.0418 | 0.1598 | 0.5165 |
| tRF | tRNA4-ThrTGT | 0.6302 | 0.3829 | 0.6667 |
| tRF | tRNA4-ValAAC | 0.1582 | 0.4648 | 0.7370 |
| tRF | tRNA4-ValTAC | 1.2560 | 0.0391 | 0.2958 |
| tRF | tRNA50-SerAGA | 0.2344 | 0.5364 | 0.7633 |
| tRF | tRNA51-SerTGA | 0.0411 | 0.8047 | 0.8539 |
| tRF | tRNA52-ArgTCT | -0.5196 | 0.1157 | 0.4653 |
| tRF | tRNA52-ProCGG | 0.4116 | 0.0967 | 0.4231 |
| tRF | tRNA54-LysTTT | 0.7547 | 0.0347 | 0.2836 |
| tRF | tRNA56-ThrTGT | -0.3755 | 0.4726 | 0.7385 |
| tRF | tRNA57-IleAAT | 0.4478 | 0.4222 | 0.6979 |
| tRF | tRNA58-LeuCAA | 0.4360 | 0.2398 | 0.5777 |
| tRF | tRNA59-GluCTC | -0.1211 | 0.6328 | 0.8121 |
| tRF | tRNA59-IleAAT | 0.8613 | 0.1216 | 0.4698 |
| tRF | tRNA5-AspGTC | -0.7443 | 0.1385 | 0.4940 |
| tRF | tRNA5-CysGCA | -1.1670 | 0.0032 | 0.0588 |
| tRF | tRNA5-GluTTC | 0.6029 | 0.0434 | 0.3117 |
| tRF | tRNA5-GlyGCC | 0.0384 | 0.7430 | 0.8416 |
| tRF | tRNA5-IleGAT | 1.2288 | 0.1190 | 0.4658 |
| tRF | tRNA5-LysCTT | -0.3886 | 0.5649 | 0.7696 |
| tRF | tRNA5-LysTTT | 0.6846 | 0.0840 | 0.3967 |
| tRF | tRNA5-SerAGA | 0.1564 | 0.6111 | 0.8000 |
| tRF | tRNA5-ValAAC | 0.1294 | 0.5379 | 0.7633 |
| tRF | tRNA61-MetCAT | -0.2549 | 0.5393 | 0.7633 |
| tRF | tRNA62-LysTTT | 0.6144 | 0.0824 | 0.3967 |
| tRF | tRNA62-SerGCT | -0.6462 | 0.0525 | 0.3333 |
| tRF | tRNA64-GluTTC | 0.3887 | 0.4867 | 0.7494 |
| tRF | tRNA65-AlaAGC | -0.4377 | 0.2998 | 0.6293 |
| tRF | tRNA65-ProAGG | 0.7478 | 0.0159 | 0.2353 |
| tRF | tRNA66-AlaTGC | 0.3959 | 0.2281 | 0.5749 |
| tRF | tRNA67-AlaAGC | -0.0654 | 0.7827 | 0.8466 |
| tRF | tRNA67-LeuCAG | 0.9864 | 0.0600 | 0.3587 |
| tRF | tRNA68-AlaAGC | -0.8914 | 0.0098 | 0.1786 |
| tRF | tRNA68-GlyGCC | -0.0350 | 0.7811 | 0.8466 |
| tRF | tRNA69-AspGTC | -0.8018 | 0.0342 | 0.2769 |
| tRF | tRNA6-AlaAGC | -1.4876 | 0.0065 | 0.1250 |
| tRF | tRNA6-AspGTC | -0.7948 | 0.1558 | 0.5138 |
| tRF | tRNA6-IleGAT | 1.4449 | 0.0754 | 0.3818 |
| tRF | tRNA6-ProCGG | 0.3329 | 0.2074 | 0.5747 |
| tRF | tRNA6-ProTGG | 0.6936 | 0.0296 | 0.2742 |
| tRF | tRNA6-PseudoCTT | -1.6229 | 0.0093 | 0.1786 |
| tRF | tRNA6-TrpCCA | 0.3676 | 0.2393 | 0.5777 |
| tRF | tRNA6-ValCAC | 0.2249 | 0.3584 | 0.6667 |
| tRF | tRNA6-ValTAC | 0.4027 | 0.3384 | 0.6608 |
| tRF | tRNA70-AlaCGC | -0.0746 | 0.7856 | 0.8466 |
| tRF | tRNA70-GlyTCC | -0.3943 | 0.3980 | 0.6807 |
| tRF | tRNA71-GluCTC | 0.0114 | 0.8509 | 0.8677 |
| tRF | tRNA71-LysTTT | -0.8955 | 0.1161 | 0.4653 |
| tRF | tRNA72-AspGTC | -0.6846 | 0.0383 | 0.2899 |
| tRF | tRNA73-GlyTCC | -0.2779 | 0.5211 | 0.7633 |
| tRF | tRNA74-GluCTC | -0.2780 | 0.3961 | 0.6798 |
| tRF | tRNA74-LeuCAA | 0.9467 | 0.0522 | 0.3333 |
| tRF | tRNA75-AspGTC | -0.9708 | 0.0055 | 0.1250 |
| tRF | tRNA76-GlyTCC | 0.7287 | 0.1773 | 0.5579 |
| tRF | tRNA76-LysTTT | 0.6514 | 0.0669 | 0.3725 |
| tRF | tRNA77-GluCTC | 0.0568 | 0.7588 | 0.8466 |
| tRF | tRNA78-AspGTC | -0.8018 | 0.0168 | 0.2368 |
| tRF | tRNA78-LeuAAG | 0.4032 | 0.2526 | 0.5885 |
| tRF | tRNA79-GlyTCC | 0.8081 | 0.3759 | 0.6667 |
| tRF | tRNA7-AsnGTT | 0.2884 | 0.3813 | 0.6667 |
| tRF | tRNA7-CysGCA | -0.6066 | 0.2944 | 0.6293 |
| tRF | tRNA7-GlnCTG | -0.7218 | 0.1830 | 0.5641 |
| tRF | tRNA7-HisGTG | 0.5084 | 0.0635 | 0.3684 |
| tRF | tRNA7-IleGAT | 1.0962 | 0.0434 | 0.3117 |
| tRF | tRNA7-LeuAAG | 0.3694 | 0.2325 | 0.5749 |
| tRF | tRNA7-LeuCAG | 1.3140 | 0.0327 | 0.2742 |
| tRF | tRNA7-LysCTT | 1.0084 | 0.0143 | 0.2258 |
| tRF | tRNA7-SerGCT | -0.0050 | 0.8580 | 0.8693 |
| tRF | tRNA80-GluCTC | 0.2298 | 0.3706 | 0.6667 |
| tRF | tRNA80-IleAAT | 0.2608 | 0.4937 | 0.7506 |
| tRF | tRNA81-AspGTC | -0.7753 | 0.0345 | 0.2836 |
| tRF | tRNA83-AsnGTT | 0.5681 | 0.2933 | 0.6293 |
| tRF | tRNA83-LeuTAA | 0.4687 | 0.2380 | 0.5777 |
| tRF | tRNA84-GluTTC | 0.2519 | 0.2352 | 0.5749 |
| tRF | tRNA85-ValCAC | 0.0103 | 0.8340 | 0.8659 |
| tRF | tRNA87-GluCTC | 0.2308 | 0.3918 | 0.6732 |
| tRF | tRNA88-PseudoCCT | 0.9229 | 0.2067 | 0.5747 |
| tRF | tRNA8-AlaTGC | 0.1041 | 0.6904 | 0.8340 |
| tRF | tRNA8-CysGCA | 0.3852 | 0.3381 | 0.6608 |
| tRF | tRNA8-HisGTG | 0.5037 | 0.0732 | 0.3818 |
| tRF | tRNA8-ProTGG | 0.5688 | 0.0429 | 0.3108 |
| tRF | tRNA8-SeCTCA | 0.1909 | 0.5389 | 0.7633 |
| tRF | tRNA8-SerGCT | 0.1925 | 0.5523 | 0.7691 |
| tRF | tRNA90-ValCAC | -0.0354 | 0.7422 | 0.8416 |
| tRF | tRNA91-PseudoCCC | 0.1120 | 0.5627 | 0.7691 |
| tRF | tRNA94-GluTTC | 0.2503 | 0.2232 | 0.5749 |
| tRF | tRNA96-PseudoCCT | 1.2148 | 0.0325 | 0.2742 |
| tRF | tRNA98-LeuAAG | 0.5051 | 0.0848 | 0.3967 |
| tRF | tRNA98-ValCAC | 0.0362 | 0.7403 | 0.8416 |
| tRF | tRNA99-GlnCTG | -0.2836 | 0.2874 | 0.6228 |
| tRF | tRNA99-ValCAC | -0.0936 | 0.6081 | 0.7974 |
| tRF | tRNA9-ArgTCT | 0.6902 | 0.2487 | 0.5853 |
| tRF | tRNA9-HisGTG | 0.4012 | 0.2604 | 0.6000 |
| tRF | tRNA9-IleAAT | 0.4365 | 0.3815 | 0.6667 |
| tRF | tRNA9-LysCTT | 0.4822 | 0.1502 | 0.5000 |
| tRF | tRNA9-ProAGG | 0.7748 | 0.0204 | 0.2609 |
| tRF | tRNA9-PseudoAAT | 0.0429 | 0.8365 | 0.8659 |
| tRF | tRNA9-ValCAC | 0.4021 | 0.2056 | 0.5747 |
| snRNA | U1.4 | -1.2569 | 0.0788 | 0.3964 |
| snRNA | U1.77 | -0.1549 | 0.5312 | 0.7633 |
| snRNA | U1.81 | -0.3761 | 0.3863 | 0.6667 |
| snRNA | U1.82 | -0.9405 | 0.0265 | 0.2742 |
| snRNA | U1.88 | -0.3076 | 0.3603 | 0.6667 |
| snRNA | U1.90 | 0.5654 | 0.2470 | 0.5837 |
| snRNA | U2.12 | -0.7577 | 0.1285 | 0.4838 |
| snRNA | U2.13 | -0.1633 | 0.6274 | 0.8093 |
| snRNA | U2.14 | -0.4204 | 0.1336 | 0.4940 |
| snRNA | U2.15 | -0.8069 | 0.0517 | 0.3333 |
| snRNA | U2.22 | -0.1201 | 0.5410 | 0.7633 |
| snRNA | U2.23 | -0.3313 | 0.2384 | 0.5777 |
| snRNA | U2.26 | -0.7119 | 0.7084 | 0.8416 |
| snRNA | U2.27 | -1.3408 | 0.0034 | 0.1111 |
| snRNA | U2.32 | -4.9307 | 0.0004 | 0.0000 |
| snRNA | U2.34 | -0.6457 | 0.1818 | 0.5641 |
| snRNA | U2.38 | -0.2700 | 0.3048 | 0.6357 |
| snRNA | U2.41 | -1.4155 | 0.0829 | 0.3967 |
| snRNA | U2.44 | 0.0822 | 0.7706 | 0.8466 |
| snRNA | U2.53 | -0.8348 | 0.5351 | 0.7633 |
| snRNA | U2.59 | -0.2804 | 0.2676 | 0.6015 |
| snRNA | U2.61 | 0.6630 | 0.2019 | 0.5747 |
| snRNA | U2.6 | -0.3814 | 0.2253 | 0.5749 |
| snRNA | U2.7 | -0.4642 | 0.1579 | 0.5138 |
| snRNA | U2.9 | -0.3048 | 0.4048 | 0.6899 |
| snRNA | U8.2 | 0.0416 | 0.8595 | 0.8693 |
| piRNA | uc021ybm.1 | -1.0809 | 0.1789 | 0.5602 |
| piRNA | uc022aol.1 | 0.6701 | 0.0886 | 0.4098 |
| YRF | Y_RNA.10 | 1.2691 | 0.0024 | 0.0588 |
| YRF | Y_RNA.105 | 0.4960 | 0.1549 | 0.5138 |
| YRF | Y_RNA.110 | 0.2444 | 0.4242 | 0.6979 |
| YRF | Y_RNA.11 | 0.6589 | 0.0929 | 0.4109 |
| YRF | Y_RNA.118 | -0.0715 | 0.7312 | 0.8416 |
| YRF | Y_RNA.119 | 0.2111 | 0.4715 | 0.7385 |
| YRF | Y_RNA.121 | -0.0469 | 0.7691 | 0.8466 |
| YRF | Y_RNA.122 | -0.6492 | 0.1393 | 0.4940 |
| YRF | Y_RNA.125 | -0.1609 | 0.5317 | 0.7633 |
| YRF | Y_RNA.128 | -0.4870 | 0.0755 | 0.3818 |
| YRF | Y_RNA.130 | -0.1974 | 0.3726 | 0.6667 |
| YRF | Y_RNA.13 | 0.7422 | 0.3815 | 0.6667 |
| YRF | Y_RNA.133 | -0.0378 | 0.7778 | 0.8466 |
| YRF | Y_RNA.134 | -0.4425 | 0.3670 | 0.6667 |
| YRF | Y_RNA.144 | -0.2398 | 0.5279 | 0.7633 |
| YRF | Y_RNA.147 | 0.7165 | 0.0328 | 0.2769 |
| YRF | Y_RNA.148 | -0.6042 | 0.0848 | 0.3967 |
| YRF | Y_RNA.158 | -0.9410 | 0.2087 | 0.5747 |
| YRF | Y_RNA.161 | -0.0839 | 0.7635 | 0.8466 |
| YRF | Y_RNA.16 | 0.5608 | 0.3786 | 0.6667 |
| YRF | Y_RNA.166 | -0.6268 | 0.1644 | 0.5272 |
| YRF | Y_RNA.168 | -0.7781 | 0.1009 | 0.4318 |
| YRF | Y_RNA.170 | -0.6391 | 0.0803 | 0.3967 |
| YRF | Y_RNA.171 | 0.5438 | 0.3402 | 0.6608 |
| YRF | Y_RNA.180 | -0.1915 | 0.3431 | 0.6614 |
| YRF | Y_RNA.182 | -0.7046 | 0.1386 | 0.4940 |
| YRF | Y_RNA.184 | -1.5507 | 0.0164 | 0.2368 |
| YRF | Y_RNA.190 | 0.4503 | 0.3245 | 0.6515 |
| YRF | Y_RNA.19 | 0.2178 | 0.4521 | 0.7199 |
| YRF | Y_RNA.197 | 0.3842 | 0.2612 | 0.6000 |
| YRF | Y_RNA.201 | 0.5168 | 0.2151 | 0.5749 |
| YRF | Y_RNA.20 | 0.8067 | 0.0650 | 0.3725 |
| YRF | Y_RNA.202 | -0.6106 | 1.0000 | 0.8693 |
| YRF | Y_RNA.207 | 0.1632 | 0.6606 | 0.8241 |
| YRF | Y_RNA.212 | -0.2823 | 0.2115 | 0.5747 |
| YRF | Y_RNA.218 | 0.4402 | 0.2035 | 0.5747 |
| YRF | Y_RNA.234 | 0.2309 | 0.6033 | 0.7939 |
| YRF | Y_RNA.24 | 0.6116 | 0.2088 | 0.5747 |
| YRF | Y_RNA.250 | -0.0748 | 0.6544 | 0.8210 |
| YRF | Y_RNA.254 | -0.1152 | 0.6846 | 0.8330 |
| YRF | Y_RNA.255 | -0.1207 | 0.5408 | 0.7633 |
| YRF | Y_RNA.256 | -0.0750 | 0.7847 | 0.8466 |
| YRF | Y_RNA.257 | -0.4360 | 0.3365 | 0.6608 |
| YRF | Y_RNA.263 | 0.7676 | 0.1146 | 0.4653 |
| YRF | Y_RNA.265 | -0.4377 | 0.4578 | 0.7285 |
| YRF | Y_RNA.273 | 0.0576 | 0.7467 | 0.8416 |
| YRF | Y_RNA.276 | -0.4400 | 0.2633 | 0.6000 |
| YRF | Y_RNA.280 | -0.0027 | 0.8677 | 0.8693 |
| YRF | Y_RNA.282 | 0.2358 | 0.6199 | 0.8084 |
| YRF | Y_RNA.289 | 0.5547 | 0.2144 | 0.5749 |
| YRF | Y_RNA.292 | -0.0341 | 0.7838 | 0.8466 |
| YRF | Y_RNA.295 | 0.1103 | 0.7004 | 0.8356 |
| YRF | Y_RNA.298 | -0.3642 | 0.4713 | 0.7385 |
| YRF | Y_RNA.30 | -0.3103 | 0.4269 | 0.6979 |
| YRF | Y_RNA.307 | 0.4106 | 0.0699 | 0.3750 |
| YRF | Y_RNA.31 | 1.2167 | 0.0160 | 0.2353 |
| YRF | Y_RNA.312 | 0.6754 | 0.1459 | 0.4942 |
| YRF | Y_RNA.315 | -0.1267 | 0.6919 | 0.8340 |
| YRF | Y_RNA.3 | -0.0293 | 0.8073 | 0.8542 |
| YRF | Y_RNA.321 | -0.0467 | 0.7556 | 0.8446 |
| YRF | Y_RNA.322 | -0.1931 | 0.5797 | 0.7747 |
| YRF | Y_RNA.324 | -0.0252 | 0.8379 | 0.8659 |
| YRF | Y_RNA.325 | 0.3419 | 0.3353 | 0.6608 |
| YRF | Y_RNA.341 | 0.0139 | 0.8445 | 0.8668 |
| YRF | Y_RNA.349 | -1.4396 | 0.0002 | 0.0000 |
| YRF | Y_RNA.353 | -0.5308 | 0.2556 | 0.5932 |
| YRF | Y_RNA.359 | -0.0641 | 0.7482 | 0.8416 |
| YRF | Y_RNA.367 | 0.2380 | 0.5537 | 0.7691 |
| YRF | Y_RNA.37 | -0.7949 | 0.0186 | 0.2500 |
| YRF | Y_RNA.384 | -0.3478 | 0.2329 | 0.5749 |
| YRF | Y_RNA.388 | 0.3334 | 0.3143 | 0.6481 |
| YRF | Y_RNA.392 | 0.1922 | 0.6387 | 0.8121 |
| YRF | Y_RNA.394 | -1.0436 | 0.0241 | 0.2708 |
| YRF | Y_RNA.396 | -0.0966 | 0.6649 | 0.8265 |
| YRF | Y_RNA.397 | -0.8411 | 0.0632 | 0.3684 |
| YRF | Y_RNA.401 | -0.2771 | 0.5482 | 0.7691 |
| YRF | Y_RNA.410 | -0.0746 | 0.7476 | 0.8416 |
| YRF | Y_RNA.412 | -0.2464 | 0.4894 | 0.7500 |
| YRF | Y_RNA.413 | -0.4907 | 0.2698 | 0.6022 |
| YRF | Y_RNA.44 | 0.0621 | 0.7601 | 0.8466 |
| YRF | Y_RNA.445 | 0.0619 | 0.7452 | 0.8416 |
| YRF | Y_RNA.450 | -0.0005 | 0.8732 | 0.8693 |
| YRF | Y_RNA.452 | 0.9020 | 0.1135 | 0.4653 |
| YRF | Y_RNA.453 | -0.1324 | 0.7462 | 0.8416 |
| YRF | Y_RNA.469 | -0.1874 | 0.5896 | 0.7821 |
| YRF | Y_RNA.470 | 0.1016 | 0.6993 | 0.8356 |
| YRF | Y_RNA.477 | -0.0856 | 0.6976 | 0.8356 |
| YRF | Y_RNA.478 | 0.1314 | 0.5859 | 0.7812 |
| YRF | Y_RNA.479 | 1.1329 | 0.0448 | 0.3117 |
| YRF | Y_RNA.481 | -0.4242 | 0.3269 | 0.6515 |
| YRF | Y_RNA.483 | 0.7371 | 0.1366 | 0.4940 |
| YRF | Y_RNA.486 | -0.1631 | 0.4743 | 0.7391 |
| YRF | Y_RNA.489 | -0.1296 | 0.7444 | 0.8416 |
| YRF | Y_RNA.492 | 0.0794 | 0.7298 | 0.8416 |
| YRF | Y_RNA.493 | -0.1407 | 0.6347 | 0.8121 |
| YRF | Y_RNA.501 | 0.4299 | 0.4717 | 0.7385 |
| YRF | Y_RNA.502 | 0.7621 | 0.1423 | 0.4940 |
| YRF | Y_RNA.505 | -0.3578 | 0.2636 | 0.6000 |
| YRF | Y_RNA.508 | 0.6876 | 0.1440 | 0.4941 |
| YRF | Y_RNA.511 | -0.2405 | 0.3796 | 0.6667 |
| YRF | Y_RNA.51 | 0.2282 | 0.4936 | 0.7506 |
| YRF | Y_RNA.519 | -0.4039 | 0.1483 | 0.5000 |
| YRF | Y_RNA.52 | -0.5599 | 0.1215 | 0.4698 |
| YRF | Y_RNA.523 | 0.6823 | 0.3836 | 0.6667 |
| YRF | Y_RNA.526 | 0.4626 | 0.1996 | 0.5747 |
| YRF | Y_RNA.53 | -1.7447 | 0.0019 | 0.0588 |
| YRF | Y_RNA.535 | -0.5070 | 0.1932 | 0.5747 |
| YRF | Y_RNA.540 | 0.5754 | 0.1847 | 0.5663 |
| YRF | Y_RNA.544 | -1.2498 | 0.0012 | 0.0000 |
| YRF | Y_RNA.549 | 2.1678 | 0.0031 | 0.0588 |
| YRF | Y_RNA.553 | -0.0875 | 0.8020 | 0.8519 |
| YRF | Y_RNA.565 | -0.9330 | 0.0005 | 0.0000 |
| YRF | Y_RNA.57 | 0.5383 | 0.2767 | 0.6065 |
| YRF | Y_RNA.572 | -0.1379 | 0.7234 | 0.8416 |
| YRF | Y_RNA.595 | 0.3175 | 0.3076 | 0.6395 |
| YRF | Y_RNA.597 | -0.1866 | 0.5609 | 0.7691 |
| YRF | Y_RNA.599 | -0.1475 | 0.6346 | 0.8121 |
| YRF | Y_RNA.601 | 0.5167 | 0.2169 | 0.5749 |
| YRF | Y_RNA.60 | -0.4284 | 0.0296 | 0.2742 |
| YRF | Y_RNA.604 | 0.3436 | 0.6664 | 0.8269 |
| YRF | Y_RNA.618 | -0.4297 | 0.3813 | 0.6667 |
| YRF | Y_RNA.619 | -0.5239 | 0.0928 | 0.4109 |
| YRF | Y_RNA.620 | 0.1371 | 0.7143 | 0.8416 |
| YRF | Y_RNA.622 | 0.6660 | 0.2450 | 0.5837 |
| YRF | Y_RNA.623 | -0.0207 | 0.8325 | 0.8659 |
| YRF | Y_RNA.630 | 1.5771 | 0.0120 | 0.2000 |
| YRF | Y_RNA.632 | -0.3457 | 0.3146 | 0.6481 |
| YRF | Y_RNA.637 | 0.8632 | 0.0755 | 0.3818 |
| YRF | Y_RNA.662 | -0.3354 | 0.3545 | 0.6667 |
| YRF | Y_RNA.663 | -0.2318 | 0.4266 | 0.6979 |
| YRF | Y_RNA.666 | 0.1750 | 0.4235 | 0.6979 |
| YRF | Y_RNA.668 | 0.0596 | 0.7863 | 0.8466 |
| YRF | Y_RNA.670 | -0.1625 | 0.5561 | 0.7691 |
| YRF | Y_RNA.673 | 0.3448 | 0.2181 | 0.5749 |
| YRF | Y_RNA.676 | 0.3924 | 0.5175 | 0.7633 |
| YRF | Y_RNA.687 | -0.1620 | 0.5850 | 0.7807 |
| YRF | Y_RNA.692 | -0.1953 | 0.3831 | 0.6667 |
| YRF | Y_RNA.696 | 0.2498 | 0.5357 | 0.7633 |
| YRF | Y_RNA.699 | 0.0312 | 0.8426 | 0.8668 |
| YRF | Y_RNA.703 | 0.1308 | 0.7795 | 0.8466 |
| YRF | Y_RNA.704 | -0.2337 | 0.4420 | 0.7124 |
| YRF | Y_RNA.709 | -0.5178 | 0.2348 | 0.5749 |
| YRF | Y_RNA.71 | -0.0382 | 0.7840 | 0.8466 |
| YRF | Y_RNA.714 | 1.0645 | 0.0694 | 0.3725 |
| YRF | Y_RNA.7 | 0.7585 | 0.0703 | 0.3750 |
| YRF | Y_RNA.725 | -0.2107 | 0.4432 | 0.7124 |
| YRF | Y_RNA.731 | -0.5182 | 0.0912 | 0.4109 |
| YRF | Y_RNA.738 | 0.0751 | 0.7663 | 0.8466 |
| YRF | Y_RNA.741 | -0.2177 | 0.4949 | 0.7506 |
| YRF | Y_RNA.745 | 0.6718 | 0.1614 | 0.5246 |
| YRF | Y_RNA.747 | -0.5100 | 0.2866 | 0.6228 |
| YRF | Y_RNA.756 | 0.3669 | 0.3194 | 0.6500 |
| YRF | Y_RNA.761 | 0.1889 | 0.6496 | 0.8210 |
| YRF | Y_RNA.766 | 0.2718 | 0.3403 | 0.6608 |
| YRF | Y_RNA.778 | -0.3797 | 0.5154 | 0.7633 |
| YRF | Y_RNA.781 | -1.8132 | 0.0125 | 0.2000 |
| YRF | Y_RNA.787 | 0.2065 | 0.5525 | 0.7691 |
| YRF | Y_RNA.789 | -0.4900 | 0.1568 | 0.5138 |
| YRF | Y_RNA.790 | -0.6691 | 0.0687 | 0.3725 |
| YRF | Y_RNA.795 | -0.5595 | 0.0407 | 0.3056 |
| YRF | Y_RNA.796 | -0.0335 | 0.8096 | 0.8547 |
| YRF | Y_RNA.800 | 0.0138 | 0.8514 | 0.8677 |
| YRF | Y_RNA.88 | 0.3959 | 0.4201 | 0.6979 |
| YRF | Y_RNA.9 | -0.5125 | 0.1247 | 0.4770 |

**Supplementary Table 1.** List of all circulating sncRNAs (612) identified by NGS in serum from children with food-mediated anaphylaxis. FC: fold change (anaphylaxis/control). Positive values indicate an increase (red) during anaphylaxis, while negative values imply a decrease (blue) during the reaction. FDR: false discovery ratio.
