# Supplementary material for: Circulating serum profile of small non-coding RNAs in patients with anaphylaxis beyond microRNAs
Source: Front Allergy. 2024 Feb 7;5:1307880. doi: 10.3389/falgy.2024.1307880 (PMC10879566; doi:10.3389/falgy.2024.1307880)
Supplement: Supplementary file 2 [file Table2.docx]

**SUPPLEMENTARY MATERIAL**

**SUPPLEMENTARY TABLE 2**

| **Group** | **sncRNA** | **FC** | **p value** | **FDR** |
| --- | --- | --- | --- | --- |
| snRNA | RNU1-10P | 0,2099 | 0,5511 | 0,8659 |
| snRNA | RNU1-11P | 0,8492 | 0.2031 | 0.8658 |
| snRNA | RNU11 | 0.4837 | 0.5293 | 0.8659 |
| snRNA | RNU1-1 | -0.0676 | 0.7785 | 0.8659 |
| snRNA | RNU1-12P | -0.0016 | 0.8637 | 0.8659 |
| snRNA | RNU1-13P | -0.8545 | 0.0362 | 0.7241 |
| snRNA | RNU12 | 0.0278 | 0.8430 | 0.8659 |
| snRNA | RNU1-2 | 0.1910 | 0.6866 | 0.8659 |
| snRNA | RNU1-22P | 0.7602 | 0.1340 | 0.8609 |
| snRNA | RNU1-3 | -0.7482 | 0.1099 | 0.8609 |
| snRNA | RNU1-4 | -0.3228 | 0.4650 | 0.8659 |
| snRNA | RNU1-5 | -0.7022 | 0.1432 | 0.8609 |
| snRNA | RNU1-6 | 0.0377 | 0.8371 | 0.8659 |
| snRNA | RNU1-7 | -0.1052 | 0.7445 | 0.8659 |
| snRNA | RNU1-8 | -0.3162 | 0.6063 | 0.8659 |
| snRNA | RNU1-9 | -0.2684 | 0.6367 | 0.8659 |
| snRNA | RNU2-2 | 0.3043 | 0.5068 | 0.8659 |
| snRNA | RNU2-3P | -0.8116 | 0.1058 | 0.8609 |
| snRNA | RNU2-4P | -0.5793 | 0.4337 | 0.8659 |
| snRNA | RNU2-5P | -0.5277 | 0.3987 | 0.8659 |
| snRNA | RNU2-6P | -0.3601 | 0.4163 | 0.8659 |
| snRNA | RNU2-7P | -0.3845 | 0.3249 | 0.8659 |
| snRNA | RNU5A-1 | 0.1879 | 0.7060 | 0.8659 |
| snRNA | RNU5B-1 | -0.4074 | 0.4867 | 0.8659 |
| snoRNA | SCARNA15 | 0.6816 | 0.1574 | 0.8609 |
| snoRNA | SCARNA16 | 1.0749 | 0.1244 | 0.8609 |
| snoRNA | SCARNA17 | -0.9137 | 0.1129 | 0.8609 |
| snoRNA | SCARNA18.2 | 0.6807 | 0.2733 | 0.8658 |
| snoRNA | SCARNA2 | -2.1143 | 0.0046 | 0.4286 |
| snoRNA | SCARNA3 | 0.5424 | 0.1590 | 0.8621 |
| snoRNA | SCARNA5 | 0.5277 | 1.0000 | 0.8659 |
| snoRNA | SCARNA6 | -0.1002 | 0.8173 | 0.8659 |
| snoRNA | SCARNA8 | 0.3974 | 0.5033 | 0.8659 |
| snoRNA | SNORA3 | 0.4617 | 0.0440 | 0.7500 |
| snoRNA | SNORA33 | 1.1168 | 0.2549 | 0.8658 |
| snoRNA | SNORA51 | -0.2507 | 0.6862 | 0.8659 |
| snoRNA | SNORA63.6 | -0.7568 | 0.3041 | 0.8658 |
| snoRNA | SNORA63 | -0.0749 | 0.7518 | 0.8659 |
| snoRNA | SNORA64 | -0.3761 | 1.0000 | 0.8659 |
| snoRNA | SNORA73B | -0.0381 | 0.8436 | 0.8659 |
| snoRNA | SNORD100 | -0.3877 | 0.6788 | 0.8659 |
| snoRNA | SNORD102 | 0.3378 | 0.5069 | 0.8659 |
| snoRNA | SNORD104 | 0.0759 | 0.7927 | 0.8659 |
| snoRNA | SNORD110 | -1.4990 | 1.0000 | 0.8659 |
| snoRNA | SNORD118 | -0.6214 | 0.4399 | 0.8659 |
| snoRNA | SNORD119 | -0.3805 | 0.4709 | 0.8659 |
| snoRNA | SNORD12C | -0.8104 | 1.0000 | 0.8659 |
| snoRNA | SNORD15A | -0.9081 | 1.0000 | 0.8659 |
| snoRNA | SNORD1C | -0.0330 | 0.8194 | 0.8659 |
| snoRNA | SNORD2.2 | -0.1224 | 1.0000 | 0.8659 |
| snoRNA | SNORD20 | -0.2381 | 0.6302 | 0.8659 |
| snoRNA | SNORD22 | 0.1281 | 0.7781 | 0.8659 |
| snoRNA | SNORD24 | -0.8431 | 0.3175 | 0.8659 |
| snoRNA | SNORD25 | -0.9673 | 0.3465 | 0.8659 |
| snoRNA | SNORD26 | 0.5251 | 0.2202 | 0.8658 |
| snoRNA | SNORD30 | -0.9321 | 0.5406 | 0.8659 |
| snoRNA | SNORD31.1 | 0.8261 | 0.2767 | 0.8658 |
| snoRNA | SNORD31 | -1.4773 | 0.3499 | 0.8659 |
| snoRNA | SNORD32A | -0.3791 | 1.0000 | 0.8659 |
| snoRNA | SNORD33 | -0.0302 | 0.8023 | 0.8659 |
| snoRNA | SNORD38A | -0.3505 | 0.7403 | 0.8659 |
| snoRNA | SNORD38B | -1.6837 | 0.2138 | 0.8658 |
| snoRNA | SNORD3A | -0.5184 | 0.2013 | 0.8658 |
| snoRNA | SNORD3B-1 | -0.4810 | 0.3001 | 0.8658 |
| snoRNA | SNORD3B-2 | -0.5008 | 0.2395 | 0.8658 |
| snoRNA | SNORD3C | -0.2934 | 0.4371 | 0.8659 |
| snoRNA | SNORD3D | -0.5892 | 0.2149 | 0.8658 |
| snoRNA | SNORD41 | -0.7450 | 0.0188 | 0.6250 |
| snoRNA | SNORD43 | -2.0565 | 1.0000 | 0.8659 |
| snoRNA | SNORD45A | -0.8839 | 0.4480 | 0.8659 |
| snoRNA | SNORD47 | 0.4593 | 1.0000 | 0.8659 |
| snoRNA | SNORD50A | -0.9770 | 0.0177 | 0.6250 |
| snoRNA | SNORD50B | -0.9142 | 0.3631 | 0.8659 |
| snoRNA | SNORD51 | 0.0836 | 0.8444 | 0.8659 |
| snoRNA | SNORD52 | -0.4970 | 0.5044 | 0.8659 |
| snoRNA | SNORD57.1 | 0.6058 | 0.5332 | 0.8659 |
| snoRNA | SNORD6 | 0.4727 | 0.7257 | 0.8659 |
| snoRNA | SNORD62A | -1.1847 | 0.3903 | 0.8659 |
| snoRNA | SNORD62B | -1.0980 | 0.4838 | 0.8659 |
| snoRNA | SNORD63 | -1.3727 | 1.0000 | 0.8659 |
| snoRNA | SNORD66 | 0.2140 | 0.8256 | 0.8659 |
| snoRNA | SNORD68 | -0.1892 | 1.0000 | 0.8659 |
| snoRNA | SNORD69 | 0.3476 | 0.6806 | 0.8659 |
| snoRNA | SNORD71 | -1.1543 | 0.3541 | 0.8659 |
| snoRNA | SNORD74 | -1.4937 | 0.3377 | 0.8659 |
| snoRNA | SNORD76 | 0.3714 | 0.7253 | 0.8659 |
| snoRNA | SNORD82 | -0.0351 | 0.8483 | 0.8659 |
| snoRNA | SNORD83A | 0.0032 | 0.8623 | 0.8659 |
| snoRNA | SNORD83B | 0.1211 | 0.7260 | 0.8659 |
| snoRNA | SNORD84-203 | -1.1480 | 0.4115 | 0.8659 |
| snoRNA | SNORD85 | -0.4762 | 0.5097 | 0.8659 |
| snoRNA | SNORD89 | -0.1180 | 0.6718 | 0.8659 |
| snoRNA | SNORD93 | -0.8240 | 0.4336 | 0.8659 |
| snoRNA | SNORD94 | 0.3346 | 0.5689 | 0.8659 |
| snoRNA | SNORD95 | 0.1154 | 0.8033 | 0.8659 |
| snoRNA | SNORD96A | -0.5310 | 1.0000 | 0.8659 |
| snoRNA | SNORD97 | 0.0961 | 0.8043 | 0.8659 |
| snoRNA | snoU2-30.1 | 0.7367 | 0.3425 | 0.8659 |
| tRF | tRNA100-LeuCAA | 0.6286 | 0.1688 | 0.8658 |
| tRF | tRNA101-AlaAGC | -0.7286 | 0.0951 | 0.8609 |
| tRF | tRNA102-AlaAGC | -0.2594 | 0.4947 | 0.8659 |
| tRF | tRNA103-AsnGTT | -0.2711 | 0.5327 | 0.8659 |
| tRF | tRNA104-AlaTGC | -0.0912 | 0.7561 | 0.8659 |
| tRF | tRNA106-HisGTG | 0.8983 | 0.0395 | 0.7333 |
| tRF | tRNA106-PheGAA | 0.6656 | 0.4608 | 0.8659 |
| tRF | tRNA107-AlaTGC | -0.5234 | 0.2999 | 0.8658 |
| tRF | tRNA107-AsnGTT | -0.0560 | 0.8053 | 0.8659 |
| tRF | tRNA108-AlaAGC | -0.4074 | 0.4444 | 0.8659 |
| tRF | tRNA108-AsnGTT | 0.5472 | 0.3504 | 0.8659 |
| tRF | tRNA109-PheGAA | 0.0439 | 1.0000 | 0.8659 |
| tRF | tRNA10-AlaCGC | -0.1464 | 0.6561 | 0.8659 |
| tRF | tRNA10-AspGTC | -0.2092 | 0.3029 | 0.8658 |
| tRF | tRNA10-CysGCA | 1.6690 | 0.2581 | 0.8658 |
| tRF | tRNA10-GlyTCC | -0.6306 | 0.2867 | 0.8658 |
| tRF | tRNA10-IleAAT | -0.6706 | 0.3185 | 0.8659 |
| tRF | tRNA10-LysCTT | 0.0967 | 0.7080 | 0.8659 |
| tRF | tRNA10-MetCAT | -0.2544 | 0.6427 | 0.8659 |
| tRF | tRNA10-PseudoCTC | 0.4555 | 0.4028 | 0.8659 |
| tRF | tRNA10-SerGCT | -0.7135 | 0.1197 | 0.8609 |
| tRF | tRNA10-ValCAC | 0.1353 | 0.4817 | 0.8659 |
| tRF | tRNA110-AlaTGC | 0.1402 | 0.7155 | 0.8659 |
| tRF | tRNA111-HisGTG | -0.1934 | 0.6679 | 0.8659 |
| tRF | tRNA112-GlnCTG | 0.5309 | 0.1028 | 0.8609 |
| tRF | tRNA113-AlaTGC | 0.3385 | 0.4730 | 0.8659 |
| tRF | tRNA115-ValAAC | 0.0404 | 0.7993 | 0.8659 |
| tRF | tRNA116-GluCTC | -0.2337 | 0.4296 | 0.8659 |
| tRF | tRNA117-GlyTCC | -0.8390 | 0.1095 | 0.8609 |
| tRF | tRNA118-HisGTG | 0.0551 | 0.7608 | 0.8659 |
| tRF | tRNA118-PseudoTTT | 0.5629 | 0.4784 | 0.8659 |
| tRF | tRNA119-AlaCGC | 0.2878 | 0.2882 | 0.8658 |
| tRF | tRNA119-LysCTT | 0.1127 | 0.6572 | 0.8659 |
| tRF | tRNA11-ArgACG | 0.7480 | 0.2202 | 0.8658 |
| tRF | tRNA11-GluTTC | -0.7252 | 0.0784 | 0.8364 |
| tRF | tRNA11-IleAAT | 0.1294 | 0.5242 | 0.8659 |
| tRF | tRNA11-LysCTT | -0.1411 | 0.5598 | 0.8659 |
| tRF | tRNA11-LysTTT | -0.0497 | 0.8174 | 0.8659 |
| tRF | tRNA11-PheGAA | 0.4755 | 0.2355 | 0.8658 |
| tRF | tRNA11-ProAGG | -0.0226 | 0.8195 | 0.8659 |
| tRF | tRNA11-SerAGA | -0.3069 | 0.1156 | 0.8609 |
| tRF | tRNA120-AlaAGC | -0.6659 | 0.1107 | 0.8609 |
| tRF | tRNA121-ThrCGT | -0.0174 | 0.8455 | 0.8659 |
| tRF | tRNA123-SerGCT | -0.5514 | 0.2134 | 0.8658 |
| tRF | tRNA126-LeuAAG | 0.1293 | 0.6422 | 0.8659 |
| tRF | tRNA127-CysGCA | 0.2408 | 0.4481 | 0.8659 |
| tRF | tRNA127-ThrTGT | 0.5056 | 0.2361 | 0.8658 |
| tRF | tRNA128-GlyGCC | 0.2040 | 0.3762 | 0.8659 |
| tRF | tRNA128-LysCTT | 0.2022 | 0.6409 | 0.8659 |
| tRF | tRNA129-MetCAT | -0.1555 | 0.6209 | 0.8659 |
| tRF | tRNA12-ArgCCT | -0.4953 | 0.4697 | 0.8659 |
| tRF | tRNA12-AspGTC | -0.2052 | 0.4003 | 0.8659 |
| tRF | tRNA12-ProAGG | 0.0070 | 0.8495 | 0.8659 |
| tRF | tRNA12-ProTGG | -0.3337 | 0.5209 | 0.8659 |
| tRF | tRNA12-PseudoGCA | -0.2712 | 0.5902 | 0.8659 |
| tRF | tRNA12-TrpCCA | -0.2835 | 0.4622 | 0.8659 |
| tRF | tRNA12-ValAAC | 0.4237 | 0.1457 | 0.8609 |
| tRF | tRNA130-GlnTTG | -0.5090 | 0.5816 | 0.8659 |
| tRF | tRNA131-GlnCTG | 0.3112 | 0.5684 | 0.8659 |
| tRF | tRNA131-GlyCCC | -0.3862 | 0.5443 | 0.8659 |
| tRF | tRNA132-ValAAC | 0.4829 | 0.1323 | 0.8609 |
| tRF | tRNA133-GlyCCC | 0.2732 | 0.1977 | 0.8658 |
| tRF | tRNA133-ValCAC | -0.4183 | 0.1319 | 0.8609 |
| tRF | tRNA134-GluTTC | -0.5788 | 0.3183 | 0.8659 |
| tRF | tRNA134-LeuTAA | -0.1927 | 0.6817 | 0.8659 |
| tRF | tRNA135-ThrAGT | 0.5336 | 0.3915 | 0.8659 |
| tRF | tRNA136-ValAAC | -0.0550 | 0.7086 | 0.8659 |
| tRF | tRNA137-Pseudo??? | -0.4981 | 0.3508 | 0.8659 |
| tRF | tRNA137-SerCGA | -0.2104 | 0.4162 | 0.8659 |
| tRF | tRNA138-ArgACG | 0.7045 | 0.1483 | 0.8609 |
| tRF | tRNA139-ValAAC | 0.1694 | 0.4256 | 0.8659 |
| tRF | tRNA13-AlaCGC | 0.0869 | 0.6368 | 0.8659 |
| tRF | tRNA13-AlaTGC | 0.0028 | 0.8589 | 0.8659 |
| tRF | tRNA13-GlyCCC | -0.2483 | 0.6385 | 0.8659 |
| tRF | tRNA13-LysCTT | 0.1724 | 0.6471 | 0.8659 |
| tRF | tRNA13-ValCAC | -0.3441 | 0.3606 | 0.8659 |
| tRF | tRNA140-LeuCAA | 1.1483 | 0.0167 | 0.6250 |
| tRF | tRNA141-LeuCAA | -0.1976 | 0.5712 | 0.8659 |
| tRF | tRNA142-MetCAT | 0.1416 | 0.4732 | 0.8659 |
| tRF | tRNA143-LysTTT | -0.0709 | 0.7948 | 0.8659 |
| tRF | tRNA144-AspGTC | -0.1299 | 0.6377 | 0.8659 |
| tRF | tRNA145-SerAGA | -0.5047 | 0.1038 | 0.8609 |
| tRF | tRNA146-GlnCTG | -0.2526 | 0.6419 | 0.8659 |
| tRF | tRNA147-SerAGA | -0.1677 | 0.4013 | 0.8659 |
| tRF | tRNA148-SerTGA | -0.4380 | 0.1812 | 0.8658 |
| tRF | tRNA149-LysTTT | 0.6710 | 0.1156 | 0.8609 |
| tRF | tRNA14-CysGCA | -0.3302 | 1.0000 | 0.8659 |
| tRF | tRNA14-LysTTT | 0.1406 | 0.5644 | 0.8659 |
| tRF | tRNA14-PheGAA | -0.7196 | 0.1549 | 0.8609 |
| tRF | tRNA14-ProTGG | 0.0825 | 0.7082 | 0.8659 |
| tRF | tRNA14-ThrCGT | -0.6789 | 0.2541 | 0.8658 |
| tRF | tRNA14-TyrGTA | 0.4758 | 0.3711 | 0.8659 |
| tRF | tRNA150-MetCAT | -0.4060 | 0.2909 | 0.8658 |
| tRF | tRNA151-ThrCGT | -0.8284 | 0.1179 | 0.8609 |
| tRF | tRNA152-ValCAC | 0.5365 | 0.2814 | 0.8658 |
| tRF | tRNA153-IleAAT | 0.6747 | 0.2617 | 0.8658 |
| tRF | tRNA154-IleAAT | -0.2074 | 0.6336 | 0.8659 |
| tRF | tRNA156-ArgACG | -0.3420 | 0.4473 | 0.8659 |
| tRF | tRNA157-ValCAC | 0.2289 | 0.3731 | 0.8659 |
| tRF | tRNA158-IleAAT | -0.0193 | 0.8273 | 0.8659 |
| tRF | tRNA15-CysGCA | 0.4434 | 0.0523 | 0.8049 |
| tRF | tRNA15-GlnCTG | 0.6954 | 0.2136 | 0.8658 |
| tRF | tRNA15-ThrCGT | 0.7080 | 1.0000 | 0.8659 |
| tRF | tRNA15-TyrGTA | -0.7730 | 0.3757 | 0.8659 |
| tRF | tRNA15-ValAAC | 0.2407 | 0.2496 | 0.8658 |
| tRF | tRNA161-AlaAGC | 0.8164 | 0.1389 | 0.8609 |
| tRF | tRNA163-IleAAT | 0.4225 | 0.4969 | 0.8659 |
| tRF | tRNA165-IleAAT | -0.1146 | 0.6101 | 0.8659 |
| tRF | tRNA166-AlaAGC | -0.3978 | 0.4306 | 0.8659 |
| tRF | tRNA168-TrpCCA | 0.2941 | 0.3271 | 0.8659 |
| tRF | tRNA169-MetCAT | 0.3233 | 0.1616 | 0.8658 |
| tRF | tRNA16-CysGCA | -0.2310 | 0.6317 | 0.8659 |
| tRF | tRNA16-GlnTTG | 1.6794 | 0.0056 | 0.4286 |
| tRF | tRNA16-HisGTG | 0.1017 | 0.6932 | 0.8659 |
| tRF | tRNA16-LeuAAG | 0.4880 | 0.1850 | 0.8658 |
| tRF | tRNA16-TyrGTA | -0.8710 | 0.1491 | 0.8609 |
| tRF | tRNA16-ValTAC | -0.2287 | 0.3701 | 0.8659 |
| tRF | tRNA171-MetCAT | 0.0911 | 0.5281 | 0.8659 |
| tRF | tRNA172-SerTGA | 0.1470 | 0.5621 | 0.8659 |
| tRF | tRNA173-GlnTTG | 0.3975 | 0.0558 | 0.8049 |
| tRF | tRNA174-GlnTTG | 0.6528 | 0.1973 | 0.8658 |
| tRF | tRNA175-SerGCT | 0.3371 | 0.4660 | 0.8659 |
| tRF | tRNA17-LeuCAG | -0.1205 | 0.7234 | 0.8659 |
| tRF | tRNA17-PseudoTTC | -0.4875 | 0.4515 | 0.8659 |
| tRF | tRNA17-SupTTA | -1.5631 | 0.0830 | 0.8448 |
| tRF | tRNA17-ValTAC | -0.2075 | 0.5696 | 0.8659 |
| tRF | tRNA18-ArgCCT | -0.5437 | 0.2322 | 0.8658 |
| tRF | tRNA18-GlyGCC | 0.0617 | 0.5754 | 0.8659 |
| tRF | tRNA18-ValCAC | 0.2566 | 0.3097 | 0.8659 |
| tRF | tRNA19-ArgTCG | -0.0821 | 0.8001 | 0.8659 |
| tRF | tRNA19-CysGCA | 0.5642 | 0.2038 | 0.8658 |
| tRF | tRNA19-GlnCTG | -0.0208 | 0.8425 | 0.8659 |
| tRF | tRNA19-GlyGCC | 0.1316 | 0.4432 | 0.8659 |
| tRF | tRNA19-LeuAAG | 0.8169 | 0.0786 | 0.8364 |
| tRF | tRNA19-TyrGTA | -0.7443 | 0.4785 | 0.8659 |
| tRF | tRNA1-AsnGTT | -0.6128 | 0.2382 | 0.8658 |
| tRF | tRNA1-GlnCTG | 0.4788 | 0.2086 | 0.8658 |
| tRF | tRNA1-HisGTG | -0.0348 | 0.8153 | 0.8659 |
| tRF | tRNA1-LeuAAG | 0.4398 | 0.1135 | 0.8609 |
| tRF | tRNA1-PheGAA | 0.0837 | 0.7427 | 0.8659 |
| tRF | tRNA1-PseudoCCC | 1.2076 | 0.0382 | 0.7241 |
| tRF | tRNA1-SeCTCA | 0.1720 | 0.4285 | 0.8659 |
| tRF | tRNA20-GluTTC | 0.4659 | 0.1830 | 0.8658 |
| tRF | tRNA20-MetCAT | -0.3682 | 0.2460 | 0.8658 |
| tRF | tRNA20-ThrTGT | -0.0265 | 0.8289 | 0.8659 |
| tRF | tRNA21-ArgCCT | -0.2324 | 0.4984 | 0.8659 |
| tRF | tRNA21-CysGCA | 0.5866 | 0.4299 | 0.8659 |
| tRF | tRNA21-HisGTG | -0.0280 | 0.8148 | 0.8659 |
| tRF | tRNA21-ThrTGT | 0.3313 | 0.3997 | 0.8659 |
| tRF | tRNA22-AspGTC | -0.3565 | 0.4866 | 0.8659 |
| tRF | tRNA22-MetCAT | 0.1007 | 0.7415 | 0.8659 |
| tRF | tRNA22-ProAGG | -0.2593 | 0.3516 | 0.8659 |
| tRF | tRNA23-ArgCCG | 0.7923 | 0.1433 | 0.8609 |
| tRF | tRNA23-LysTTT | -0.2462 | 0.6359 | 0.8659 |
| tRF | tRNA23-ProAGG | -0.2367 | 0.5973 | 0.8659 |
| tRF | tRNA24-AlaAGC | 0.8832 | 1.0000 | 0.8659 |
| tRF | tRNA24-GlyGCC | 0.1340 | 0.4702 | 0.8659 |
| tRF | tRNA25-GlyGCC | 0.2013 | 0.4066 | 0.8659 |
| tRF | tRNA25-PseudoCTC | -0.8602 | 0.1302 | 0.8609 |
| tRF | tRNA26-AsnGTT | -1.0535 | 0.1123 | 0.8609 |
| tRF | tRNA26-CysGCA | 0.8556 | 0.0654 | 0.8261 |
| tRF | tRNA26-LeuCAG | 0.4257 | 0.2598 | 0.8658 |
| tRF | tRNA27-CysGCA | -0.0027 | 0.8617 | 0.8659 |
| tRF | tRNA27-GlyCCC | 0.4149 | 0.2370 | 0.8658 |
| tRF | tRNA27-LeuTAG | 0.7383 | 0.1558 | 0.8609 |
| tRF | tRNA28-CysGCA | 0.1174 | 0.6679 | 0.8659 |
| tRF | tRNA28-GlnCTG | 0.7571 | 0.2578 | 0.8658 |
| tRF | tRNA28-IleAAT | -0.4772 | 0.0806 | 0.8421 |
| tRF | tRNA28-ProTGG | 0.2899 | 0.3343 | 0.8659 |
| tRF | tRNA29-ProAGG | 0.1794 | 0.5380 | 0.8659 |
| tRF | tRNA2-ArgCCT | 0.1793 | 0.6082 | 0.8659 |
| tRF | tRNA2-GlyCCC | -0.2668 | 0.6265 | 0.8659 |
| tRF | tRNA2-GlyGCC | -0.2434 | 0.3388 | 0.8659 |
| tRF | tRNA2-GlyTCC | 0.2227 | 0.5483 | 0.8659 |
| tRF | tRNA2-LeuTAG | 0.4660 | 0.1530 | 0.8609 |
| tRF | tRNA2-LysCTT | 0.0969 | 0.7162 | 0.8659 |
| tRF | tRNA2-LysTTT | 0.5425 | 0.0577 | 0.8049 |
| tRF | tRNA2-MetCAT | 0.0665 | 0.7052 | 0.8659 |
| tRF | tRNA2-ProAGG | -0.0019 | 0.8616 | 0.8659 |
| tRF | tRNA2-SerCGA | 0.6500 | 0.1459 | 0.8609 |
| tRF | tRNA2-SerTGA | 0.1309 | 0.5821 | 0.8659 |
| tRF | tRNA2-TyrGTA | -0.7411 | 0.2518 | 0.8658 |
| tRF | tRNA2-ValAAC | 0.2256 | 0.2331 | 0.8658 |
| tRF | tRNA2-ValCAC | 0.2913 | 0.1389 | 0.8609 |
| tRF | tRNA30-CysGCA | -0.1140 | 0.5866 | 0.8659 |
| tRF | tRNA30-LysCTT | -0.0515 | 0.8288 | 0.8659 |
| tRF | tRNA30-ProCGG | -0.1735 | 0.5645 | 0.8659 |
| tRF | tRNA31-AsnGTT | -0.7491 | 0.3688 | 0.8659 |
| tRF | tRNA31-SerGCT | -0.1620 | 0.6659 | 0.8659 |
| tRF | tRNA32-LysCTT | -0.2003 | 0.5669 | 0.8659 |
| tRF | tRNA32-MetCAT | 0.1815 | 0.4580 | 0.8659 |
| tRF | tRNA33-HisGTG | -0.2892 | 0.4528 | 0.8659 |
| tRF | tRNA33-PseudoAGG | 0.9515 | 0.0014 | 0.0000 |
| tRF | tRNA34-GlyCCC | 0.5501 | 0.1631 | 0.8658 |
| tRF | tRNA34-IleAAT | -0.3287 | 0.2573 | 0.8658 |
| tRF | tRNA34-LeuCAG | -0.3085 | 0.5829 | 0.8659 |
| tRF | tRNA35-GlyGCC | -0.2379 | 0.2907 | 0.8658 |
| tRF | tRNA35-SerAGA | -0.2025 | 0.3754 | 0.8659 |
| tRF | tRNA35-SerCGA | 0.0184 | 0.8332 | 0.8659 |
| tRF | tRNA36-ArgACG | 0.3478 | 1.0000 | 0.8659 |
| tRF | tRNA36-LeuCAG | -0.4049 | 0.4220 | 0.8659 |
| tRF | tRNA37-GlyGCC | -0.1859 | 0.4152 | 0.8659 |
| tRF | tRNA37-ProCGG | 0.2444 | 0.3139 | 0.8659 |
| tRF | tRNA37-ValAAC | 0.1355 | 0.5401 | 0.8659 |
| tRF | tRNA38-AspGTC | -0.1904 | 0.5059 | 0.8659 |
| tRF | tRNA38-LeuCAG | -0.3643 | 0.4361 | 0.8659 |
| tRF | tRNA39-GlyGCC | -0.0725 | 0.7155 | 0.8659 |
| tRF | tRNA39-PseudoAAT | 0.2314 | 0.5975 | 0.8659 |
| tRF | tRNA3-AlaAGC | 0.1411 | 0.6367 | 0.8659 |
| tRF | tRNA3-ArgCCT | 0.4695 | 0.3540 | 0.8659 |
| tRF | tRNA3-ArgTCT | 1.0497 | 0.0089 | 0.5000 |
| tRF | tRNA3-CysGCA | 0.1209 | 0.6007 | 0.8659 |
| tRF | tRNA3-GlnCTG | -0.0087 | 0.8567 | 0.8659 |
| tRF | tRNA3-GluTTC | -0.7108 | 0.0848 | 0.8475 |
| tRF | tRNA3-LeuAAG | 0.7839 | 0.0759 | 0.8364 |
| tRF | tRNA3-ProTGG | -0.2128 | 0.4064 | 0.8659 |
| tRF | tRNA3-PseudoCAC | 0.8623 | 0.1313 | 0.8609 |
| tRF | tRNA40-LeuCAG | 0.4825 | 0.4183 | 0.8659 |
| tRF | tRNA40-ThrAGT | 0.5277 | 0.2882 | 0.8658 |
| tRF | tRNA40-ValTAC | 0.2275 | 0.3216 | 0.8659 |
| tRF | tRNA41-GlyGCC | -0.1404 | 0.5549 | 0.8659 |
| tRF | tRNA41-PseudoACT | -0.6467 | 0.4814 | 0.8659 |
| tRF | tRNA41-SerCGA | -0.2682 | 0.1524 | 0.8609 |
| tRF | tRNA42-GlnCTG | -0.6757 | 0.0655 | 0.8261 |
| tRF | tRNA42-LeuCAG | -0.4962 | 0.3497 | 0.8659 |
| tRF | tRNA42-LeuTAG | -0.3469 | 0.3791 | 0.8659 |
| tRF | tRNA43-GlyGCC | -0.2375 | 0.6647 | 0.8659 |
| tRF | tRNA43-SerGCT | -0.9398 | 0.0422 | 0.7419 |
| tRF | tRNA44-SerAGA | -0.6671 | 0.0568 | 0.8049 |
| tRF | tRNA45-AspGTC | -0.2595 | 0.2110 | 0.8658 |
| tRF | tRNA45-GlyTCC | -0.0346 | 0.8209 | 0.8659 |
| tRF | tRNA46-SerAGA | -0.0388 | 0.8038 | 0.8659 |
| tRF | tRNA47-AsnGTT | -0.0023 | 0.8631 | 0.8659 |
| tRF | tRNA47-SerAGA | -0.4373 | 0.1471 | 0.8609 |
| tRF | tRNA48-AspGTC | -0.0045 | 0.8549 | 0.8659 |
| tRF | tRNA49-GlnCTG | -0.3486 | 0.6075 | 0.8659 |
| tRF | tRNA4-ArgTCG | 0.0919 | 0.7676 | 0.8659 |
| tRF | tRNA4-ArgTCT | 0.7611 | 0.0644 | 0.8261 |
| tRF | tRNA4-AsnGTT | -0.6315 | 0.4878 | 0.8659 |
| tRF | tRNA4-AspGTC | -0.0180 | 0.8206 | 0.8659 |
| tRF | tRNA4-GlyCCC | 0.0348 | 0.7123 | 0.8659 |
| tRF | tRNA4-LeuTAA | 0.7476 | 0.2871 | 0.8658 |
| tRF | tRNA4-LysCTT | 0.3653 | 0.5383 | 0.8659 |
| tRF | tRNA4-ThrTGT | 0.2843 | 0.6633 | 0.8659 |
| tRF | tRNA4-TyrGTA | -0.6125 | 0.2659 | 0.8658 |
| tRF | tRNA4-ValAAC | 0.2708 | 0.3041 | 0.8658 |
| tRF | tRNA4-ValTAC | -0.2739 | 0.5116 | 0.8659 |
| tRF | tRNA50-SerAGA | -0.2053 | 0.2708 | 0.8658 |
| tRF | tRNA51-SerTGA | -0.2591 | 0.3161 | 0.8659 |
| tRF | tRNA52-ArgTCT | -0.2925 | 0.3606 | 0.8659 |
| tRF | tRNA52-ProCGG | -0.0925 | 0.6432 | 0.8659 |
| tRF | tRNA54-LysTTT | 0.5765 | 0.0677 | 0.8364 |
| tRF | tRNA56-ThrTGT | 1.1578 | 0.0781 | 0.8364 |
| tRF | tRNA57-IleAAT | -0.1235 | 0.6363 | 0.8659 |
| tRF | tRNA58-LeuCAA | 0.1587 | 0.6573 | 0.8659 |
| tRF | tRNA59-GluCTC | -0.3904 | 0.2208 | 0.8658 |
| tRF | tRNA59-IleAAT | -0.3752 | 0.3092 | 0.8659 |
| tRF | tRNA5-AspGTC | 0.0029 | 0.8561 | 0.8659 |
| tRF | tRNA5-CysGCA | -0.7089 | 0.2026 | 0.8658 |
| tRF | tRNA5-GluTTC | -0.1388 | 0.4982 | 0.8659 |
| tRF | tRNA5-GlyGCC | 0.1140 | 0.4364 | 0.8659 |
| tRF | tRNA5-IleGAT | 0.0459 | 0.7896 | 0.8659 |
| tRF | tRNA5-LysCTT | 0.2892 | 0.5231 | 0.8659 |
| tRF | tRNA5-LysTTT | 0.3847 | 0.2450 | 0.8658 |
| tRF | tRNA5-SerAGA | -0.1693 | 0.4245 | 0.8659 |
| tRF | tRNA5-TyrGTA | -0.7180 | 0.4497 | 0.8659 |
| tRF | tRNA5-ValAAC | 0.2075 | 0.3574 | 0.8659 |
| tRF | tRNA61-MetCAT | 0.1699 | 0.4284 | 0.8659 |
| tRF | tRNA62-LysTTT | 0.3274 | 0.2503 | 0.8658 |
| tRF | tRNA62-SerGCT | -0.0685 | 0.7580 | 0.8659 |
| tRF | tRNA65-AlaAGC | -0.4893 | 0.2780 | 0.8658 |
| tRF | tRNA65-ProAGG | 0.1500 | 0.5305 | 0.8659 |
| tRF | tRNA66-AlaTGC | -0.1079 | 0.5299 | 0.8659 |
| tRF | tRNA67-AlaAGC | 0.1481 | 0.7240 | 0.8659 |
| tRF | tRNA67-LeuCAG | 0.4180 | 0.3773 | 0.8659 |
| tRF | tRNA68-AlaAGC | -0.3772 | 0.4202 | 0.8659 |
| tRF | tRNA68-GlyGCC | 0.2246 | 0.3897 | 0.8659 |
| tRF | tRNA69-AspGTC | -0.2595 | 0.2407 | 0.8658 |
| tRF | tRNA6-AlaAGC | -0.4114 | 0.3325 | 0.8659 |
| tRF | tRNA6-ArgACG | 0.0512 | 0.8434 | 0.8659 |
| tRF | tRNA6-AspGTC | 0.0603 | 0.7597 | 0.8659 |
| tRF | tRNA6-IleGAT | -0.3410 | 0.4087 | 0.8659 |
| tRF | tRNA6-ProCGG | 0.0653 | 0.7609 | 0.8659 |
| tRF | tRNA6-ProTGG | -0.0571 | 0.7440 | 0.8659 |
| tRF | tRNA6-PseudoCTT | -0.2328 | 0.5388 | 0.8659 |
| tRF | tRNA6-TrpCCA | 0.6312 | 0.1143 | 0.8609 |
| tRF | tRNA6-ValCAC | 0.2094 | 0.4201 | 0.8659 |
| tRF | tRNA6-ValTAC | -0.4350 | 0.3897 | 0.8659 |
| tRF | tRNA70-AlaCGC | -0.5775 | 0.2829 | 0.8658 |
| tRF | tRNA70-GlyTCC | -0.4074 | 0.2233 | 0.8658 |
| tRF | tRNA71-GluCTC | -0.2393 | 0.4224 | 0.8659 |
| tRF | tRNA71-LysTTT | 0.6406 | 0.5121 | 0.8659 |
| tRF | tRNA72-AspGTC | -0.1555 | 0.4520 | 0.8659 |
| tRF | tRNA73-ArgCCG | -0.9079 | 1.0000 | 0.8659 |
| tRF | tRNA73-GlyTCC | -0.3206 | 0.3913 | 0.8659 |
| tRF | tRNA74-GluCTC | -0.2389 | 0.3824 | 0.8659 |
| tRF | tRNA74-LeuCAA | 1.0105 | 0.0571 | 0.8049 |
| tRF | tRNA75-AspGTC | 0.1193 | 0.6348 | 0.8659 |
| tRF | tRNA76-GlyTCC | -0.3244 | 0.2886 | 0.8658 |
| tRF | tRNA76-LysTTT | 0.4161 | 0.2254 | 0.8658 |
| tRF | tRNA77-GluCTC | -0.3959 | 0.2556 | 0.8658 |
| tRF | tRNA78-AspGTC | -0.1934 | 0.3885 | 0.8659 |
| tRF | tRNA78-LeuAAG | 0.2576 | 0.4020 | 0.8659 |
| tRF | tRNA79-GlyTCC | -0.7519 | 0.1670 | 0.8658 |
| tRF | tRNA7-ArgACG | -0.4713 | 0.1750 | 0.8658 |
| tRF | tRNA7-AsnGTT | 0.2888 | 0.3113 | 0.8659 |
| tRF | tRNA7-CysGCA | -0.5407 | 0.3158 | 0.8659 |
| tRF | tRNA7-GlnCTG | 0.0470 | 0.8308 | 0.8659 |
| tRF | tRNA7-HisGTG | -0.1096 | 0.6829 | 0.8659 |
| tRF | tRNA7-IleGAT | -0.1283 | 0.6624 | 0.8659 |
| tRF | tRNA7-LeuAAG | 0.4205 | 0.3467 | 0.8659 |
| tRF | tRNA7-LeuCAG | 0.7374 | 0.2092 | 0.8658 |
| tRF | tRNA7-LysCTT | -0.3218 | 0.2253 | 0.8658 |
| tRF | tRNA7-SerGCT | -0.0774 | 0.7759 | 0.8659 |
| tRF | tRNA80-GluCTC | -0.3642 | 0.2341 | 0.8658 |
| tRF | tRNA80-IleAAT | 0.1127 | 0.7043 | 0.8659 |
| tRF | tRNA81-AspGTC | 0.0236 | 0.8099 | 0.8659 |
| tRF | tRNA81-LeuTAA | -1.3991 | 1.0000 | 0.8659 |
| tRF | tRNA83-AsnGTT | -0.4527 | 0.5798 | 0.8659 |
| tRF | tRNA83-LeuTAA | 0.2273 | 0.5732 | 0.8659 |
| tRF | tRNA84-GluTTC | -0.7268 | 0.2364 | 0.8658 |
| tRF | tRNA85-ValCAC | 0.2594 | 0.2291 | 0.8658 |
| tRF | tRNA87-GluCTC | -0.3207 | 0.3310 | 0.8659 |
| tRF | tRNA88-PseudoCCT | -0.9830 | 0.0941 | 0.8609 |
| tRF | tRNA8-AlaTGC | 0.1075 | 0.6595 | 0.8659 |
| tRF | tRNA8-ArgACG | 0.6136 | 0.4301 | 0.8659 |
| tRF | tRNA8-CysGCA | 0.4933 | 0.4881 | 0.8659 |
| tRF | tRNA8-HisGTG | -0.1432 | 0.6560 | 0.8659 |
| tRF | tRNA8-ProTGG | 0.1529 | 0.4733 | 0.8659 |
| tRF | tRNA8-SeCTCA | 1.0829 | 0.0656 | 0.8261 |
| tRF | tRNA8-SerGCT | -0.0965 | 0.7224 | 0.8659 |
| tRF | tRNA8-ThrAGT | -0.0122 | 0.8429 | 0.8659 |
| tRF | tRNA90-ValCAC | 0.2875 | 0.1969 | 0.8658 |
| tRF | tRNA91-PseudoCCC | -0.5317 | 0.4234 | 0.8659 |
| tRF | tRNA94-GluTTC | -0.6345 | 0.3030 | 0.8658 |
| tRF | tRNA96-PheGAA | 0.0306 | 0.8500 | 0.8659 |
| tRF | tRNA96-PseudoCCT | -0.6800 | 0.3783 | 0.8659 |
| tRF | tRNA98-LeuAAG | 0.0699 | 0.7759 | 0.8659 |
| tRF | tRNA98-ValCAC | -0.0687 | 0.6457 | 0.8659 |
| tRF | tRNA99-GlnCTG | -0.2177 | 0.6495 | 0.8659 |
| tRF | tRNA99-ValCAC | -0.3627 | 0.5471 | 0.8659 |
| tRF | tRNA9-ArgTCT | -0.2023 | 0.5435 | 0.8659 |
| tRF | tRNA9-HisGTG | 0.0642 | 0.7609 | 0.8659 |
| tRF | tRNA9-IleAAT | -0.6493 | 0.1701 | 0.8658 |
| tRF | tRNA9-LysCTT | 0.0686 | 0.7248 | 0.8659 |
| tRF | tRNA9-ProAGG | -0.3118 | 0.3330 | 0.8659 |
| tRF | tRNA9-PseudoAAT | -0.6989 | 0.2782 | 0.8658 |
| tRF | tRNA9-ValCAC | 0.2849 | 0.3155 | 0.8659 |
| snRNA | U1.17 | 0.4432 | 0.2047 | 0.8658 |
| snRNA | U1.37 | 0.7025 | 0.3663 | 0.8659 |
| snRNA | U1.58 | -0.0739 | 0.8144 | 0.8659 |
| snRNA | U1.64 | 0.0122 | 0.8519 | 0.8659 |
| snRNA | U1.77 | -1.1407 | 0.2734 | 0.8658 |
| snRNA | U1.79 | 0.5159 | 1.0000 | 0.8659 |
| snRNA | U1.81 | -0.4583 | 0.2885 | 0.8658 |
| snRNA | U1.82 | 0.0569 | 0.8197 | 0.8659 |
| snRNA | U1.83 | 0.2727 | 0.6493 | 0.8659 |
| snRNA | U1.88 | -0.2104 | 0.6656 | 0.8659 |
| snRNA | U1.90 | -0.3534 | 0.1366 | 0.8609 |
| snRNA | U2.12 | -0.1416 | 0.7187 | 0.8659 |
| snRNA | U2.13 | -0.5227 | 0.5093 | 0.8659 |
| snRNA | U2.14 | -0.4468 | 0.3009 | 0.8658 |
| snRNA | U2.15 | -0.7251 | 0.2200 | 0.8658 |
| snRNA | U2.16 | -1.2923 | 0.2411 | 0.8658 |
| snRNA | U2.22 | -0.3300 | 0.3685 | 0.8659 |
| snRNA | U2.23 | 0.2448 | 0.5073 | 0.8659 |
| snRNA | U2.27 | -0.4270 | 0.4000 | 0.8659 |
| snRNA | U2.34 | -0.5318 | 0.3806 | 0.8659 |
| snRNA | U2.38 | 0.3479 | 0.2767 | 0.8658 |
| snRNA | U2.41 | 0.5950 | 0.5352 | 0.8659 |
| snRNA | U2.44 | -0.7186 | 0.2331 | 0.8658 |
| snRNA | U2.59 | -0.2488 | 0.4255 | 0.8659 |
| snRNA | U2.61 | -0.1189 | 0.7616 | 0.8659 |
| snRNA | U2.6 | -0.2262 | 0.5177 | 0.8659 |
| snRNA | U2.7 | 0.3596 | 0.4342 | 0.8659 |
| snRNA | U2.9 | -0.3204 | 0.5109 | 0.8659 |
| snRNA | U6.436 | 1.3260 | 1.0000 | 0.8659 |
| snRNA | U6.610 | -0.8711 | 0.5785 | 0.8659 |
| piRNA | uc011ley.2 | -1.1601 | 0.0281 | 0.7241 |
| piRNA | uc021ybm.1 | 0.4580 | 0.4201 | 0.8659 |
| piRNA | uc022aol.1 | -0.1040 | 0.7886 | 0.8659 |
| YRF | Y_RNA.10 | 0.0156 | 0.8530 | 0.8659 |
| YRF | Y_RNA.105 | 0.9039 | 0.1244 | 0.8609 |
| YRF | Y_RNA.110 | 0.1865 | 0.5362 | 0.8659 |
| YRF | Y_RNA.11 | 0.8592 | 0.0760 | 0.8364 |
| YRF | Y_RNA.118 | 0.7969 | 0.0147 | 0.6250 |
| YRF | Y_RNA.119 | 0.1451 | 0.6127 | 0.8659 |
| YRF | Y_RNA.121 | 0.2618 | 0.4064 | 0.8659 |
| YRF | Y_RNA.122 | 0.8824 | 0.0475 | 0.7879 |
| YRF | Y_RNA.125 | 0.4783 | 0.1442 | 0.8609 |
| YRF | Y_RNA.128 | 0.7047 | 1.0000 | 0.8659 |
| YRF | Y_RNA.130 | 0.2049 | 0.4946 | 0.8659 |
| YRF | Y_RNA.13 | 0.2330 | 0.4186 | 0.8659 |
| YRF | Y_RNA.133 | 0.3349 | 0.3618 | 0.8659 |
| YRF | Y_RNA.134 | -0.2884 | 0.5118 | 0.8659 |
| YRF | Y_RNA.144 | 0.1506 | 0.6742 | 0.8659 |
| YRF | Y_RNA.147 | -0.0216 | 0.8373 | 0.8659 |
| YRF | Y_RNA.148 | 0.2725 | 0.4730 | 0.8659 |
| YRF | Y_RNA.150 | 1.3130 | 0.0229 | 0.6842 |
| YRF | Y_RNA.161 | 0.0459 | 0.8207 | 0.8659 |
| YRF | Y_RNA.16 | 0.0691 | 0.7715 | 0.8659 |
| YRF | Y_RNA.163 | -0.0117 | 0.8481 | 0.8659 |
| YRF | Y_RNA.166 | -0.0130 | 0.8436 | 0.8659 |
| YRF | Y_RNA.168 | 0.1519 | 0.6556 | 0.8659 |
| YRF | Y_RNA.170 | 0.2177 | 0.4651 | 0.8659 |
| YRF | Y_RNA.171 | 0.6355 | 0.1704 | 0.8658 |
| YRF | Y_RNA.180 | -0.9568 | 0.1379 | 0.8609 |
| YRF | Y_RNA.182 | 0.0735 | 0.6513 | 0.8659 |
| YRF | Y_RNA.183 | -0.0390 | 0.6539 | 0.8659 |
| YRF | Y_RNA.190 | 0.2758 | 0.5222 | 0.8659 |
| YRF | Y_RNA.19 | 0.0310 | 0.7891 | 0.8659 |
| YRF | Y_RNA.197 | -0.2589 | 0.5874 | 0.8659 |
| YRF | Y_RNA.201 | 1.2777 | 0.0294 | 0.7241 |
| YRF | Y_RNA.20 | 0.0661 | 0.7981 | 0.8659 |
| YRF | Y_RNA.202 | 0.1920 | 0.7151 | 0.8659 |
| YRF | Y_RNA.205 | -0.9102 | 0.2451 | 0.8658 |
| YRF | Y_RNA.207 | 0.9492 | 0.1881 | 0.8658 |
| YRF | Y_RNA.212 | 0.5956 | 0.2730 | 0.8658 |
| YRF | Y_RNA.218 | 0.3361 | 0.1887 | 0.8658 |
| YRF | Y_RNA.231 | 0.8339 | 1.0000 | 0.8659 |
| YRF | Y_RNA.234 | -0.0667 | 0.7687 | 0.8659 |
| YRF | Y_RNA.24 | -0.2630 | 0.5699 | 0.8659 |
| YRF | Y_RNA.242 | 0.9286 | 1.0000 | 0.8659 |
| YRF | Y_RNA.246 | 0.1007 | 0.7422 | 0.8659 |
| YRF | Y_RNA.250 | 0.1021 | 0.7223 | 0.8659 |
| YRF | Y_RNA.251 | 0.4731 | 1.0000 | 0.8659 |
| YRF | Y_RNA.254 | 0.7564 | 0.1888 | 0.8658 |
| YRF | Y_RNA.255 | 0.5819 | 0.1377 | 0.8609 |
| YRF | Y_RNA.256 | 0.5552 | 0.2065 | 0.8658 |
| YRF | Y_RNA.257 | 0.1645 | 0.4056 | 0.8659 |
| YRF | Y_RNA.263 | -0.1046 | 0.7234 | 0.8659 |
| YRF | Y_RNA.265 | -0.4175 | 0.5763 | 0.8659 |
| YRF | Y_RNA.273 | 0.4522 | 0.3006 | 0.8658 |
| YRF | Y_RNA.276 | -0.8623 | 0.1779 | 0.8658 |
| YRF | Y_RNA.280 | 0.3914 | 0.1816 | 0.8658 |
| YRF | Y_RNA.282 | 0.1918 | 0.6569 | 0.8659 |
| YRF | Y_RNA.288 | 0.9103 | 0.0028 | 0.4286 |
| YRF | Y_RNA.289 | -0.8555 | 0.2832 | 0.8658 |
| YRF | Y_RNA.292 | 0.0314 | 0.8133 | 0.8659 |
| YRF | Y_RNA.295 | 1.3074 | 0.0095 | 0.5556 |
| YRF | Y_RNA.30 | 1.0723 | 0.0319 | 0.7241 |
| YRF | Y_RNA.307 | 0.6274 | 0.1354 | 0.8609 |
| YRF | Y_RNA.308 | 0.2734 | 0.5843 | 0.8659 |
| YRF | Y_RNA.31 | -0.3207 | 0.1866 | 0.8658 |
| YRF | Y_RNA.312 | 0.0881 | 0.7489 | 0.8659 |
| YRF | Y_RNA.315 | 0.4832 | 0.0997 | 0.8609 |
| YRF | Y_RNA.318 | -0.3908 | 0.1648 | 0.8658 |
| YRF | Y_RNA.3 | 0.1525 | 0.5330 | 0.8659 |
| YRF | Y_RNA.321 | 0.8571 | 0.0573 | 0.8049 |
| YRF | Y_RNA.322 | 0.2731 | 0.6019 | 0.8659 |
| YRF | Y_RNA.324 | -0.5677 | 0.1221 | 0.8609 |
| YRF | Y_RNA.325 | -0.2381 | 0.6778 | 0.8659 |
| YRF | Y_RNA.337 | -1.2918 | 0.0886 | 0.8609 |
| YRF | Y_RNA.340 | -0.0541 | 0.7432 | 0.8659 |
| YRF | Y_RNA.341 | 0.2910 | 0.5210 | 0.8659 |
| YRF | Y_RNA.343 | -0.1818 | 0.7100 | 0.8659 |
| YRF | Y_RNA.349 | -0.3101 | 0.6639 | 0.8659 |
| YRF | Y_RNA.353 | 0.0562 | 0.7494 | 0.8659 |
| YRF | Y_RNA.357 | 0.7595 | 0.2118 | 0.8658 |
| YRF | Y_RNA.359 | -0.1233 | 0.6775 | 0.8659 |
| YRF | Y_RNA.367 | 0.3172 | 0.5444 | 0.8659 |
| YRF | Y_RNA.37 | 0.3734 | 0.4451 | 0.8659 |
| YRF | Y_RNA.376 | 0.7561 | 0.0189 | 0.6250 |
| YRF | Y_RNA.384 | 0.0525 | 0.8169 | 0.8659 |
| YRF | Y_RNA.388 | -0.5568 | 0.2930 | 0.8658 |
| YRF | Y_RNA.389 | -0.3489 | 0.6901 | 0.8659 |
| YRF | Y_RNA.392 | 1.0104 | 0.1160 | 0.8609 |
| YRF | Y_RNA.394 | 1.2775 | 0.0068 | 0.4286 |
| YRF | Y_RNA.396 | -0.4444 | 0.2986 | 0.8658 |
| YRF | Y_RNA.397 | 0.3258 | 0.4689 | 0.8659 |
| YRF | Y_RNA.401 | -0.3844 | 0.5601 | 0.8659 |
| YRF | Y_RNA.410 | 0.1294 | 0.5774 | 0.8659 |
| YRF | Y_RNA.412 | -0.1270 | 0.7907 | 0.8659 |
| YRF | Y_RNA.413 | 0.7408 | 0.0384 | 0.7241 |
| YRF | Y_RNA.414 | 0.5612 | 0.2833 | 0.8658 |
| YRF | Y_RNA.420 | 0.4799 | 0.5660 | 0.8659 |
| YRF | Y_RNA.428 | 1.1254 | 0.0705 | 0.8364 |
| YRF | Y_RNA.44 | -0.1738 | 0.6980 | 0.8659 |
| YRF | Y_RNA.445 | 1.2116 | 0.0173 | 0.6250 |
| YRF | Y_RNA.450 | 0.1492 | 0.5854 | 0.8659 |
| YRF | Y_RNA.45 | -0.0285 | 0.8278 | 0.8659 |
| YRF | Y_RNA.452 | 0.9395 | 0.0756 | 0.8364 |
| YRF | Y_RNA.453 | 0.6121 | 0.1366 | 0.8609 |
| YRF | Y_RNA.469 | 0.1749 | 0.6954 | 0.8659 |
| YRF | Y_RNA.470 | -0.1745 | 0.4774 | 0.8659 |
| YRF | Y_RNA.476 | -0.4195 | 1.0000 | 0.8659 |
| YRF | Y_RNA.477 | 0.5367 | 0.3822 | 0.8659 |
| YRF | Y_RNA.478 | 0.5416 | 0.2223 | 0.8658 |
| YRF | Y_RNA.479 | -0.5527 | 0.3419 | 0.8659 |
| YRF | Y_RNA.481 | 0.2448 | 0.2966 | 0.8658 |
| YRF | Y_RNA.483 | 0.3463 | 0.2115 | 0.8658 |
| YRF | Y_RNA.486 | 0.4048 | 0.3799 | 0.8659 |
| YRF | Y_RNA.489 | 0.1952 | 0.6631 | 0.8659 |
| YRF | Y_RNA.49 | -1.7268 | 0.0041 | 0.4286 |
| YRF | Y_RNA.492 | 0.2712 | 0.4067 | 0.8659 |
| YRF | Y_RNA.493 | 0.1575 | 0.7329 | 0.8659 |
| YRF | Y_RNA.501 | 0.3240 | 0.4532 | 0.8659 |
| YRF | Y_RNA.502 | 0.8809 | 0.1048 | 0.8609 |
| YRF | Y_RNA.505 | 0.3433 | 0.3830 | 0.8659 |
| YRF | Y_RNA.507 | -0.1708 | 0.6303 | 0.8659 |
| YRF | Y_RNA.508 | -0.6359 | 0.1793 | 0.8658 |
| YRF | Y_RNA.510 | 0.8236 | 0.1214 | 0.8609 |
| YRF | Y_RNA.511 | 0.3988 | 0.2702 | 0.8658 |
| YRF | Y_RNA.51 | -0.1288 | 0.6528 | 0.8659 |
| YRF | Y_RNA.518 | 0.7486 | 0.1439 | 0.8609 |
| YRF | Y_RNA.519 | 0.9511 | 0.0558 | 0.8049 |
| YRF | Y_RNA.52 | 0.9242 | 0.0579 | 0.8095 |
| YRF | Y_RNA.523 | -0.3153 | 0.4583 | 0.8659 |
| YRF | Y_RNA.526 | 0.7381 | 0.0561 | 0.8049 |
| YRF | Y_RNA.535 | 0.2818 | 0.4632 | 0.8659 |
| YRF | Y_RNA.537 | 0.5704 | 0.2265 | 0.8658 |
| YRF | Y_RNA.540 | -0.1624 | 0.6703 | 0.8659 |
| YRF | Y_RNA.544 | 0.0333 | 0.8183 | 0.8659 |
| YRF | Y_RNA.549 | 0.4777 | 0.1576 | 0.8609 |
| YRF | Y_RNA.553 | 0.1823 | 0.6637 | 0.8659 |
| YRF | Y_RNA.559 | -0.3715 | 0.2870 | 0.8658 |
| YRF | Y_RNA.565 | -0.5653 | 0.4119 | 0.8659 |
| YRF | Y_RNA.57 | 0.4157 | 0.2740 | 0.8658 |
| YRF | Y_RNA.572 | 0.1679 | 0.7260 | 0.8659 |
| YRF | Y_RNA.595 | 0.1000 | 0.6097 | 0.8659 |
| YRF | Y_RNA.597 | 0.3854 | 0.3051 | 0.8659 |
| YRF | Y_RNA.599 | 0.9279 | 0.0771 | 0.8364 |
| YRF | Y_RNA.601 | 0.0117 | 0.8490 | 0.8659 |
| YRF | Y_RNA.604 | -0.0656 | 0.7276 | 0.8659 |
| YRF | Y_RNA.605 | -0.9642 | 0.0241 | 0.6842 |
| YRF | Y_RNA.618 | 0.5799 | 0.1757 | 0.8658 |
| YRF | Y_RNA.619 | 0.3416 | 0.2254 | 0.8658 |
| YRF | Y_RNA.620 | 0.1530 | 0.5011 | 0.8659 |
| YRF | Y_RNA.622 | -0.4903 | 0.5393 | 0.8659 |
| YRF | Y_RNA.623 | 0.5882 | 0.0198 | 0.6250 |
| YRF | Y_RNA.625 | 1.3868 | 0.0372 | 0.7241 |
| YRF | Y_RNA.627 | -0.4780 | 1.0000 | 0.8659 |
| YRF | Y_RNA.630 | 0.6378 | 0.1545 | 0.8609 |
| YRF | Y_RNA.632 | -0.5980 | 0.0975 | 0.8609 |
| YRF | Y_RNA.637 | -0.0070 | 0.8566 | 0.8659 |
| YRF | Y_RNA.662 | -0.7658 | 0.1059 | 0.8609 |
| YRF | Y_RNA.663 | -0.1093 | 0.7032 | 0.8659 |
| YRF | Y_RNA.666 | -0.4020 | 0.4077 | 0.8659 |
| YRF | Y_RNA.668 | 0.3032 | 0.2896 | 0.8658 |
| YRF | Y_RNA.670 | 0.2253 | 0.4777 | 0.8659 |
| YRF | Y_RNA.673 | -0.2984 | 0.5325 | 0.8659 |
| YRF | Y_RNA.676 | 0.0154 | 0.8524 | 0.8659 |
| YRF | Y_RNA.679 | 0.5648 | 0.5568 | 0.8659 |
| YRF | Y_RNA.687 | -0.0763 | 0.7260 | 0.8659 |
| YRF | Y_RNA.692 | -0.0883 | 0.7273 | 0.8659 |
| YRF | Y_RNA.696 | -0.3283 | 0.6252 | 0.8659 |
| YRF | Y_RNA.699 | 0.7219 | 0.0892 | 0.8609 |
| YRF | Y_RNA.700 | 0.8308 | 0.0375 | 0.7241 |
| YRF | Y_RNA.703 | -0.6125 | 0.2659 | 0.8658 |
| YRF | Y_RNA.704 | 0.2531 | 0.2853 | 0.8658 |
| YRF | Y_RNA.71 | -0.0057 | 0.8561 | 0.8659 |
| YRF | Y_RNA.714 | -0.4568 | 0.1480 | 0.8609 |
| YRF | Y_RNA.7 | 0.0312 | 0.8045 | 0.8659 |
| YRF | Y_RNA.725 | 0.1067 | 0.6757 | 0.8659 |
| YRF | Y_RNA.731 | 0.1016 | 0.7376 | 0.8659 |
| YRF | Y_RNA.738 | 0.0189 | 0.8461 | 0.8659 |
| YRF | Y_RNA.741 | 0.4366 | 0.2317 | 0.8658 |
| YRF | Y_RNA.745 | 0.3664 | 0.5518 | 0.8659 |
| YRF | Y_RNA.756 | 0.2605 | 0.4968 | 0.8659 |
| YRF | Y_RNA.761 | 1.2393 | 0.0063 | 0.4286 |
| YRF | Y_RNA.764 | 0.2953 | 0.6826 | 0.8659 |
| YRF | Y_RNA.766 | -0.1210 | 0.6526 | 0.8659 |
| YRF | Y_RNA.778 | 0.6528 | 0.0317 | 0.7241 |
| YRF | Y_RNA.781 | 0.4731 | 0.0386 | 0.7241 |
| YRF | Y_RNA.787 | 0.7507 | 0.0796 | 0.8393 |
| YRF | Y_RNA.789 | -0.3540 | 0.4439 | 0.8659 |
| YRF | Y_RNA.790 | -0.2421 | 0.2133 | 0.8658 |
| YRF | Y_RNA.796 | -0.0578 | 0.6994 | 0.8659 |
| YRF | Y_RNA.800 | -0.2556 | 0.6715 | 0.8659 |
| YRF | Y_RNA.807 | 0.7957 | 0.1664 | 0.8658 |
| YRF | Y_RNA.88 | -0.1527 | 0.5947 | 0.8659 |
| YRF | Y_RNA.90 | -1.8527 | 0.0241 | 0.6842 |
| YRF | Y_RNA.9 | 0.2282 | 0.5692 | 0.8659 |

**Supplementary Table 2.** List of all circulating sncRNAs (671) identified by NGS in serum from adults with drug-mediated anaphylaxis. FC: fold change (anaphylaxis/control). Positive values indicate an increase (red) during anaphylaxis, while negative values imply a decrease (blue) during the reaction. FDR: false discovery ratio.
